# RNase L restricts the mobility of engineered retrotransposons in cultured human cells

**DOI:** 10.1093/nar/gkt1308

**Published:** 2013-12-25

**Authors:** Ao Zhang, Beihua Dong, Aurélien J. Doucet, John B. Moldovan, John V. Moran, Robert H. Silverman

**Affiliations:** ^1^Department of Molecular Medicine, Cleveland Clinic Lerner College of Medicine of Case Western Reserve University, Cleveland Clinic, Cleveland, OH, 44195, USA, ^2^Department of Cancer Biology, Lerner Research Institute, Cleveland Clinic, Cleveland, OH 44195, USA, ^3^Department of Human Genetics, Ann Arbor, MI 48109, USA, ^4^Cellular and Molecular Biology Program, Ann Arbor, MI 48109, USA, ^5^Department of Internal Medicine, Ann Arbor, MI 48109, USA and ^6^Howard Hughes Medical Institute, University of Michigan Medical School, Ann Arbor, Michigan, 48109, USA

## Abstract

Retrotransposons are mobile genetic elements, and their mobility can lead to genomic instability. Retrotransposon insertions are associated with a diverse range of sporadic diseases, including cancer. Thus, it is not a surprise that multiple host defense mechanisms suppress retrotransposition. The 2′,5′-oligoadenylate (2-5A) synthetase (OAS)-RNase L system is a mechanism for restricting viral infections during the interferon antiviral response. Here, we investigated a potential role for the OAS-RNase L system in the restriction of retrotransposons. Expression of wild type (WT) and a constitutively active form of RNase L (NΔ385), but not a catalytically inactive RNase L mutant (R667A), impaired the mobility of engineered human LINE-1 (L1) and mouse intracisternal A-type particle retrotransposons in cultured human cells. Furthermore, WT RNase L, but not an inactive RNase L mutant (R667A), reduced L1 RNA levels and subsequent expression of the L1-encoded proteins (ORF1p and ORF2p). Consistently, confocal immunofluorescent microscopy demonstrated that WT RNase L, but not RNase L R667A, prevented formation of L1 cytoplasmic foci. Finally, siRNA-mediated depletion of endogenous RNase L in a human ovarian cancer cell line (Hey1b) increased the levels of L1 retrotransposition by ∼2-fold. Together, these data suggest that RNase L might function as a suppressor of structurally distinct retrotransposons.

## INTRODUCTION

Transposable elements comprise at least 45 and 37.5% of the human and mouse genomes, respectively (1,2). They are classified by whether they replicate via a DNA (transposons) or an RNA intermediate (retrotransposons) [reviewed in (3)]. DNA transposons originally were discovered in maize as mutable loci capable of mobilizing to new genomic locations (3,4). DNA transposons comprise ∼3% of the human genome (1) and were active during primate evolution until ∼37 million years ago (5). However, with the exception of certain bat species (6), DNA transposons appear to be inactive in most mammalian genomes (1).

Unlike the ‘cut-and-paste’ mobility mechanism used by DNA transposons, retrotransposons mobilize via a ‘copy-and-paste’ mechanism that uses an RNA intermediate [reviewed in (7)]. There are two major groups of retrotransposons that are distinguishable by the presence or absence of long terminal repeats (LTRs). LTR-retrotransposons include human endogenous retroviruses (HERVs) as well as murine intracisternal A-particle (IAP) and MusD sequences [reviewed in (8–10)]. Endogenous LTR-retrotransposons are structurally similar to retroviruses, but generally lack or contain a defective envelope (*env*) gene, which relegates them to intracellular replication [reviewed in (11)]. While HERVs appear to be inactive in the human genome, it is estimated that ∼300 copies of IAP and 10 copies of MusD remain functional in the mouse genome (12–14).

Non-LTR retrotransposons, including Long INterspersed Element-1 (LINE-1 or L1) and the Small INterspersed Elements (SINEs), account for about one-third of human genomic DNA (1). L1 elements are the only class of autonomously active human retrotransposons [reviewed in (10)]. The L1-encoded proteins (ORF1p and/or ORF2p) can also mobilize certain SINEs (e.g. Alu and SVA elements) (10,15–17). Importantly, L1-mediated retrotransposition events continue to cause insertional mutagenesis and genetic disorders in humans [reviewed in (18)].

**Figure 1.**
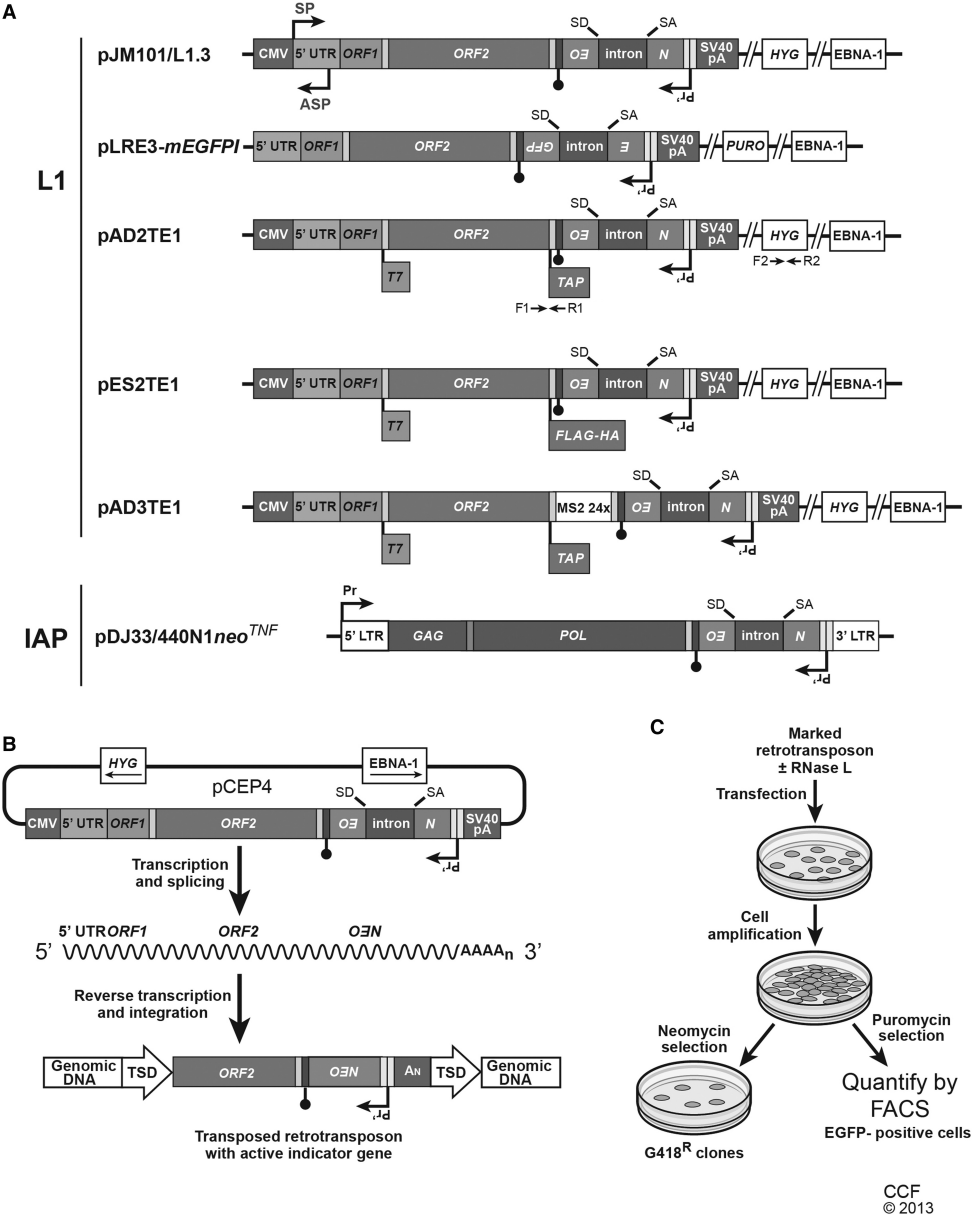
An overview of the L1 and IAP retrotransposition assays. (**A**) Schematics of L1 and IAP constructs: The L1 and IAP constructs contain a NEO-based (*mneoI*) or *EGFP*-based (*mEGFPI*) retrotransposition indicator cassette near their 3′ ends. The indicator cassettes are in an anti-sense (backward) orientation relative to the transcriptional orientation of the L1 or IAP elements. The indicator cassettes also contain an intron that is in the same transcriptional orientation as the retroelement. SD and SA indicate the splice donor and splice acceptor sites of the intron, respectively. Pr′ indicates the promoter driving the expression of the retrotransposition indicator cassette. Closed lollipops indicate the polyadenylation signal on the indicator cassette. A CMV promoter enhances the expression of the pJM101/L1.3, pAD2TE1, pES2TE1 and pAD3TE1 L1 vectors. An SV40 polyadenylation signal is present at the 3′ end of each L1 expression cassette. Notably, the *mneoI*-based L1 vectors are expressed from a pCEP4 vector that contains a HYG and an EBNA-1 gene. The *mEGFPI-based* L1 vectors are expressed from a pCEP4 vector that was modified to contain a PURO gene; it also contains the EBNA-1 gene. Flag symbols indicate the names of epitope-tags present in some L1 vectors. The SP and ASP labels indicate the sense and anti-sense promoters located in the L1 5′-UTR. The MS2 24x designation indicates the 24 copies of the MS2-GFP RNA binding motif in the pAD3TE1 construct. The PCR primers for pAD2TE1 are labeled F1, R1, F2 and R2 (see ‘Materials and Methods’ section for details). In the IAP vector [pDJ33/440N1*neo^TNF^* (13)], Pr indicates the viral LTR promoter. The IAP GAG and POL genes also are indicated. (**B**) Rationale of the assay: Transcription from a promoter driving L1 or IAP expression allows splicing of the intron from either the *mneoI-* or *EGFP-based* indicator cassettes. Retrotransposition of the resultant RNA leads to activation of the reporter gene, conferring either G418-resistance or EGFP-positivity to host cells. TSD indicates a target site duplication flanking the retrotransposed L1. (**C**) Experimental protocols to detect L1 retrotransposition: Cells were co-transfected with an engineered L1 or IAP retroelement and either an empty vector (pFLAG-CMV-2) or amino-terminal FLAG-tagged RNase L expression plasmid. For the *mneoI*-based assays, the transfected cells were subjected to G418 selection 2 days after transfection. The numbers of G418-resistant foci serve as a readout of retrotransposition efficiency. For the *mEGFPI*-based assays, FACS analysis was used to measure the percentage of EGFP-positive cells 4 days after transfection (See ‘Materials and Methods’ section for further details about each assay).

Human L1 sequences represent ∼17% of human genomic DNA (1). While the vast majority of L1s are molecular fossils incapable of retrotransposition, it is estimated that ∼80–100 L1s remain retrotransposition-competent (19,20). Full-length human L1s are ∼6 kb in length (21,22). They contain an internal sense-strand promoter (SP) located within their 5′-untranslated region (UTR) (23–27). Transcription generates a bicistronic mRNA that consists of the L1 5′-UTR, two open reading frames (ORF1 and ORF2) and a 3′-UTR that ends in a poly (A) tail (21,22). The L1 5′-UTR also contains an antisense promoter (ASP), which drives the production of an RNA containing a region of the 5′-UTR conjoined to an mRNA sequence derived from genomic sequences located upstream of the L1 integration site (28) [see pJM101/L1.3 gene map in Figure 1A (top)].

Human ORF1 encodes an ∼40 kDa RNA binding protein (ORF1p) with nucleic acid chaperone activity (29-31). ORF2 encodes an ∼150 kDa protein (ORF2p) that contains both endonuclease (32) and reverse transcriptase (33) activities. ORF1p and ORF2p preferentially bind to their encoding L1 mRNA by a process termed *cis*-preference (34–36), which leads to the formation of an L1 ribonucleoprotein particle (RNP) (30,37,38). The resultant L1 RNPs are transported into the nucleus, where L1 integration is completed by target-site primed reverse transcription (32,39,40). The activities associated with ORF1p and ORF2p are both required for efficient L1 retrotransposition (41).

L1 retrotransposition can lead to local genomic rearrangements (e.g. deletions and inversions) at their integration sites [reviewed in (10)]. Moreover, L1 retrotransposition events may influence the expression of genes near the integration sites [reviewed in (10)]. Thus far, 96 L1-mediated retrotransposition events have been reported to be responsible for a wide range of single-gene diseases in humans [reviewed in (18)]. In addition, ORF2p may generate double-strand breaks in genomic DNA, which have the potential to be mutagenic (42,43).

The host cell has evolved various strategies to regulate retrotransposon activity at both the transcriptional and posttranscriptional levels [reviewed in (7)]. For example, retrotransposon-derived Piwi interacting RNAs, in conjunction with Piwi proteins, can degrade L1 and other transposable element transcripts in the germ line of mice and flies, and they are thought to be involved in the epigenetic silencing of retrotransposons via promoter methylation in murine embryonic male germ cells [reviewed in (44,45)]. It also is proposed that hybridization of the L1 sense and antisense transcripts may serve as double-strand RNA triggers for Dicer-dependent RNA interference mediated regulation of L1 retrotransposition (46), although this supposition requires further study. Besides these small RNA-based inhibition pathways, L1 retrotransposition can be inhibited by several proteins, including the apolipoprotein B mRNA editing enzyme 3 (APOBEC3) family of cytidine deaminases [reviewed in (47)], Trex1 (48) and MOV10 (49–51). Recent evidence also suggests the ataxia telangiectasia mutated protein may limit the length and/or number of engineered L1 retrotransposition events in cultured cells (52). In addition, heterogeneous nuclear ribonucleoprotein L (hnRNPL) binds L1 RNA and interferes with L1 retrotransposition (53,54). HnRNPL and several other cellular inhibitors of L1 retrotransposition were also identified in the L1 ORF1 protein interactome (54). In contrast, the poly(A) binding protein C1 was recently shown to promote L1 retrotransposition (55).

The interferon (IFN) regulated 2′,5′-oligoadenylate (2-5A) synthetase (OAS)-RNase L system inhibits viral replication, but it is unclear whether it restricts retrotransposon activity [reviewed in (56)]. The OAS genes encode IFN inducible enzymes that are expressed at basal levels in many mammalian cell types (57). Viral dsRNAs activate OAS-1, -2 and -3, which use ATP to generate 2-5A molecules with the following structures: [p_x_5′A(2′p5′A)_n_; x = 1–3; n ≥ 2] (58). The 2-5A then binds to the ankyrin repeat domain of latent RNase L, causing it to form an enzymatically active dimer (59). Active RNase L cleaves single-strand regions of viral and cellular RNA, suppressing viral protein synthesis, replication and spread [reviewed in (56)]. Moreover, cleavage products generated by RNase L, mostly short duplex RNAs with 3′-phosphoryl groups, can bind and activate the RIG-I and MDA5 helicases (60). Interaction of these helicases with the mitochondrial adapter MAVS then results in a signaling cascade, allowing type I IFN production (60). The prolonged activation of RNase L results in cell death through apoptosis, leading to the elimination of virus-infected cells (61–63).

The antiviral activity of the OAS-RNase L pathway combats the infectivity of numerous RNA and DNA viruses [reviewed in (56)]. Here we demonstrate that wild type (WT) RNase L and a constitutively active (NΔ385) RNase L mutant potently restrict both L1 and IAP retrotransposition in cultured human cells. In contrast, RNase L (R667A), catalytically inactive due to a mutation in the active site, does not restrict L1 or IAP retrotransposition. Consistent with the above observations, siRNA-mediated knockdown of endogenous RNase L leads to a ∼2-fold increase in L1 retrotransposition. Finally, the expression of active forms of RNase L, but not the R667A RNase L mutant, leads to the degradation of L1 mRNA, which, in turn, leads to a decrease in the expression of L1 ORF1p and ORF2p. Thus, in addition to its role in restricting the infectivity of several viruses, RNase L may act to restrict the retrotransposition of certain endogenous retrotransposons.

## MATERIALS AND METHODS

### Plasmid constructs

Schematic maps of L1 and IAP plasmids used in this study are shown (Figure 1A and B and Supplementary Figures S4A and S5A). Brief descriptions of each plasmid used in this study and the original references describing the plasmid construction are provided below.

**pCEP4:** is a mammalian expression vector (Gibco/Life Technologies/InVitrogen) used to construct some of the L1 plasmids (as indicated below) and contains a cytomegalovirus (CMV) promoter and an SV40 polyadenylation signal. The plasmid backbone also contains a hygromycin resistance gene (HYG) and the Epstein Barr Virus Nuclear Antigen-1 gene (EBNA-1).

**pJM101/L1.3:** is a pCEP4-based plasmid that contains an active human L1 (L1.3) equipped with an *mneoI* retrotransposition indicator cassette (20,64).

**pLRE3-*mEGFPI*:** is a pCEP4-based plasmid that contains an active human L1 (LRE3) equipped with an *mEGFPI* retrotransposition indicator cassette (65). The pCEP4 backbone was modified to contain a puromycin resistance (PURO) gene in place of the HYG. The CMV promoter also was deleted from the vector; thus, L1 expression is only driven by its native 5′-UTR (65).

**pAD2TE1:** is a pCEP4-based plasmid similar to pJM101/L1.3. It was modified to contain a T7 *gene10* epitope-tag on the carboxyl-terminus of ORF1p and a TAP epitope-tag on the carboxyl-terminus of ORF2p. Its 3′-UTR contains the *mneol* retrotransposition indicator cassette (66).

**pES2TE1:** is identical to pAD2TE1, but was modified to replace the TAP tag on the carboxyl-terminus of ORF2p with a FLAG-HA tag (66).

**pAD3TE1:** is identical to pAD2TE1, but was modified to contain 24 copies of the MS2 stem-loop RNA binding repeats upstream of the *mneoI* indicator cassette (66).

**pDJ33/440N1*neo^TNF^*:** is a gift from Thierry Heidmann (Institut Gustave Roussy, Paris, France). It contains a mouse IAP tagged with a neomycin resistance gene (NEO) retrotransposition indicator cassette similar to the one present in pJM101/L1.3 (13).

**pJM111-LRE3-*mEGFPI*:** is identical to pLRE3-*mEGFPI*, but contains two missense mutations in ORF1p (RR261-262AA), which render the L1 retrotransposition-defective (41). Mr William Giblin (University of Michigan) made the plasmid.

**pDK500:** is a pCEP4-based ORF1 expression plasmid. It contains the L1.3 5′-UTR, ORF1 containing a T7 *gene 10* epitope-tag at its carboxyl-terminus and the *mneoI* retrotransposition cassette (38).

**pAD500:** is a pCEP4-based ORF2 expression plasmid. It contains the L1.3 5′-UTR, ORF2 containing a TAP epitope-tag at its carboxyl-terminus and the *mneoI* retrotransposition cassette (66).

**pMS2-GFP:** was obtained from Addgene (plasmid 27121), and was originally deposited by Robert Singer (67). It encodes a nuclear localized MS2-GFP fusion protein.

The human HA epitope-tagged APOBEC3A (A3A) expression plasmid was obtained from Dr Bryan Cullen at Duke University (68). The A3A cDNA was subcloned into pFLAG-CMV-2 (Sigma-Aldrich) to ensure that it was expressed from the same context as the RNase L constructs used in this study.

The human cDNAs for RNase L (69), A3A and RIG-I (a gift from Michael Gale, Seattle, WA, USA) were cloned into pFLAG-CMV-2 (Sigma Aldrich). They all contain a FLAG tag at their amino terminus**.** Plasmid pIREShyg (Clontech) contains a hygromycin B phosphotransferease gene under control of a CMV promoter and downstream of an internal ribosome entry site from encephalomyocarditis virus.

The catalytically inactive (R667A) RNase L mutant (70) was generated by site-directed mutagenesis and verified by DNA sequence analysis. The constitutively active (NΔ385) RNase L mutant was described in the same study (70).

Myc-tagged WT and mutant RNase L cDNAs were cloned into a modified pcDNA 3.0 (Gibco/Life Technologies/InVitrogen) vector that lacks a NEO using standard molecular cloning protocols. Briefly, the plasmids were double digested with *Bst*BI and *Sfo*I, followed by 3′ end filling with Klenow fragment of DNA polymerase, and blunt-end cloned into the modified pcDNA 3.0 vector using T4 DNA ligase (New England BioLabs).

### Cells and culture media

HeLa-M cells, which are deficient for RNase L (71), and Hey1b cells (a human ovarian cancer cell line that was a gift from Alexander Marks, University of Toronto, Toronto, Canada) (72) were maintained in Dulbecco's modified Eagle's medium (DMEM) and RPMI medium, respectively. The complete medium was supplemented with 10% fetal bovine serum (FBS), 50 U/ml of penicillin, 50 µg/ml of streptomycin and 2 mM l-glutamine (Gibco/Life Technologies).

### Retrotransposition assays

Retrotransposition assays were performed as described previously with minor modifications (65,73). Briefly, for G418-resistance–based retrotransposition assays, HeLa-M cells (∼8 × 10^4^ per well) were seeded into two sets of six-well plates. The next day, the cells were co-transfected with 0.5 µg of the indicated L1 or IAP expression plasmid and 0.5 µg of a corresponding expression plasmid for RNase L, A3A, RIG-I or an empty vector (pFLAG-CMV-2) using 3 µl of the Fugene6 transfection reagent (Roche) per well. Forty-eight hours after transfection, the cells were collected from one set of plates and were analyzed for protein expression in western blot experiments. Cells from the other set of plates were trypsinized and resuspended in complete DMEM medium supplemented with G418 (500 µg/ml) (Gibco/Life Technologies). Cells from each well were split into three 10-cm tissue culture dishes, generating triplicate cultures. After 10 days of G418 selection, the remaining cells were treated with 10% neutral buffered formalin for 5 min to fix them to tissue culture plates and then were stained with 0.05% crystal violet for 30 min to facilitate their visualization. The dishes were washed in phosphate buffered saline (PBS), scanned and foci numbers were determined using Integrated Colony Enumerator software (National Institute of Standards and Technology) (74). Notably, toxicity control reactions were performed in a similar manner (in triplicate). Briefly HeLa-M cells were co-transfected with 0.5 µg of the pcDNA 3.0 NEO expression vector and 0.5 µg of the RNase L expression plasmids using 3 µl of the Fugene6 transfection reagent per well (Roche). After G418 selection (500 µg/ml) for 10 days, the remaining cells were fixed, stained and counted.

For enhanced green fluorescent protein (EGFP)-based retrotransposition assays, HeLa-M cells were transfected with 0.5 µg of an active (pLRE3-*mEGFPI*) or inactive (pJM111-LRE3-*mEGFPI*) L1 expression plasmid and 0.5 µg of a corresponding RNase L expression plasmid, using 3 µl of Fugene6 transfection reagent (Roche) per well. The transfected cells then were subjected to puromycin selection (1 µg/ml) (Gibco/Life Technologies) to enrich for cells containing the L1 expression plasmids. After 4 days, the cells from each well were detached with a nonenzymatic cell dissociation solution (Cellgro), washed with PBS containing 1% FBS and analyzed on a FACScan (Becton-Dickinson) without fixation. For each sample, 2 × 10^5^ cells were analyzed. Data were analyzed with FlowJo software (TreeStar Inc.).

In experiments to study the effect of endogenous RNase L on L1 retrotransposition, Hey1b cells (4 × 10^5^ cells/well) were plated in a six-well tissue culture dish. The next day, the cells were transfected with 50 nM of a control siRNA pool (sc-37007, Santa Cruz Biotechnology) or an siRNA pool against RNase L (sc-45965, Santa Cruz Biotechnology) using the DharmaFECT 1 transfection reagent (Thermo Scientific). Twenty-four hours later, the cells in each well were transfected with pLRE3-*mEGFPI* or pJM111-LRE3-*mEGFPI* (1 µg), using 3 µl of the Fugene6 transfection reagent (Roche). After another 12 h, cells were trypsinized and counted as noted above. An aliquot of the cells (one-tenth) was used to monitor the endogenous RNase L protein level. The remaining cells were replated (one well was split into three wells to generate triplicate technical replicates) and were subjected to 4 days of puromycin selection (1 µg/ml, Gibco/Life Technologies). After 4 days of puromycin selection (5 and 6 days after transfection with L1 construct and siRNA, respectively), the percentage of GFP positive cells was determined by flow cytometry as described above.

### Preparation of L1 RNPs, total cell lysates and western blot assays

L1 RNPs were isolated as described previously (38) with some modifications. Briefly, HeLa-M cells were plated into two identical sets of six-well plates. The next day, the cells in each well were co-transfected with 0.5 µg of an engineered L1 expression construct (pAD2TE1, pDK500 or pAD500) and 0.5 µg of a corresponding RNase L plasmid (FLAG-WT RNase L, FLAG-RNase L R667A or FLAG-RNase L NΔ385) using 3 µl of the Fugene6 transfection reagent (Roche). The cells in one set of plates were harvested 48 h after transfection, and RNase L expression was monitored using an RNase L monoclonal antibody by western blot. Cells from the other set of plates were replated into a 10-cm dish and were subjected to selection in DMEM medium supplemented with hygromycin (200 µg/ml) (Gibco/Life Technologies) for four additional days to detect L1 protein expression 6 days after transfection. The remaining cells then were harvested and were resuspended in 1 ml of lysis buffer (20 mM HEPES, pH 7.5; 1.5 mM KCl; 2.5 mM MgCl_2_; 0.5% NP-40) containing complete mini EDTA-free protease inhibitor cocktail (Roche) per 0.5 ml of packed cell volume. After incubation on ice for 10 min, cell lysates were centrifuged at 3000 × g for 10 min at 4°C to remove cell debris. Protein concentrations were determined with Bradford assays (Biorad). One-fiftieth of the supernatants (∼50 µg of total protein) was used for protein analysis (total cell lysates in Figure 8 and Supplementary Figures S4 and S5). Aliquots of total cell lysate (∼150 µg) were ultracentrifuged at 160 000 × g for 90 min to concentrate the L1 RNP fraction. After ultracentrifugation, the supernatants were removed, the pellets were resuspended with 50 µl 1 × sodium dodecyl sulphate-polyacrylamide gel electrophoresis sample buffer (Novagen) and 20 µl were used for western blot analysis (RNP fractions in Figure 8 and Supplementary Figures S4 and S5).

For control experiments, an EGFP-encoding plasmid, pEGFP-C1 (Clontech), was co-transfected with a corresponding RNase L expression plasmid (WT, R667A and NΔ385) into HeLa-M cells. Total cell lysates were prepared 48 h after transfection (as described above) and were analyzed in western blots.

In general, western blots were developed using the ECL substrate (GE Healthcare) and exposed to autoradiography film (Denville Scientific). The Western Bright ECL HRP Substrate (Advansta) was used to detect ORF1p expressed from the pAD2TE1 and pDK500 expression constructs, as well as ORF2p from the pAD500 expression construct. The SuperSignal West Pico Chemiluminescent Substrate (Pierce) was used to detect ORF2p expressed from pAD2TE1 construct.

The following antibodies were used in western blotting experiments: mouse anti-T7 (1:5000 dilution, Novagen), rabbit anti-TAP (1:1000 dilution, Open Biosystems), rabbit anti-S6 (1:2000 dilution, Cell Signaling Technology), mouse anti-FLAG M2 (1:5000 dilution, Sigma Aldrich), mouse anti-β-actin (1:50 000 dilution, Sigma Aldrich), mouse anti-GFP (1:5000 dilution, Santa Cruz), rabbit anti-GAPDH (1:2000 dilution, Cell Signaling Technology) and mouse anti-RNase L (1:2000 dilution) (59). HRP-linked goat anti-mouse and anti-rabbit secondary antibodies were purchased from Cell Signaling Technology and were used at 1:2000 dilutions.

### Quantitative real time polymerase chain reaction to detect L1 RNA

HeLa-M cells were co-transfected with 0.5 µg of pAD2TE1 and 0.5 µg of one of the following vectors: pFLAG-CMV-2 empty vector, FLAG-tagged WT RNase L or FLAG-tagged RNase L R667A. Forty-eight hours later, total RNA was prepared with Trizol (Gibco/Life Technologies) according to manufacturer’s protocol. After contaminating DNA was removed using a Turbo DNA-free kit (Ambion), cDNA was synthesized using the High Capacity cDNA Reverse Transcription (RT) kit (Gibco/Life Technologies). The resultant cDNA was amplified using Sybr Green PCR master mix (Gibco/Life Technologies) on a StepOnePlus system according to manufacturer’s protocol. Primers were designed to amplify a 91-bp fragment specific to pAD2TE1 mRNA. The product spanned the junction of L1 ORF2 gene and the coding sequences of the engineered TAP epitope-tag in pAD2TE1 (Figure 1A).

The following primers were used to detect L1 RNA from pAD2TE1:

Forward primer (F1), 5′-ACACCGCATATTCCCACTCATAG-3′; reverse primer (R1), 5′-GCGGTTGGCTGCTGAGAC-3′.
The following primers were used to detect the HYG mRNA from the pCEP4 backbone: forward primer (F2), 5′-CAGCGAGAGCCTGACCTATTG-3′; reverse primer (R2), 5′-CAGGCAGGTCTTGCAACGT-3′.

All the primers were designed with Primer Express 3.0 software (Applied Biosystems) and data were analyzed with the 2^-ΔΔCT^ method (75).

### Immunofluorescence

Immunofluorescence experiments to detect the co-expression of the L1 and RNase L proteins were performed as described previously with minor modifications (66). Briefly, HeLa-M cells (8 × 10^4^) were plated onto sterile glass cover slips in each well of six-well tissue culture plates. The next day, adherent cells were co-transfected with 1 µg of pES2TE1 and 1 µg of one of the following constructs: an empty vector (pcDNA 3.0) (Gibco/Life Technologies/InVitrogen), a Myc-tagged WT RNase L or a Myc-tagged catalytically inactive RNase L mutant (R667A), using 6 µl of the Fugene6 transfection reagent. Forty-eight hours after transfection, the cells were fixed with freshly prepared 4% paraformaldehyde (Electron Microscopy Sciences) in 1 × PBS for 10 min at room temperature. The fixed cells were washed three times with PBS and permeabilized by treatment with ice-cold anhydrous methanol for 1 min at −20°C. After another 1 × PBS wash, cells were blocked by incubation with 3% goat serum and 0.1% Triton X-100 in 1 × PBS for 1 h at room temperature. The permeabilized cells then were incubated with primary antibodies overnight at 4°C in a humidified chamber. The cells were washed three times with 1 × PBS and incubated with secondary antibodies for 1 h at 37°C in the dark. After four 1 × PBS washes, the coverslips were mounted with Vectashield mounting media with DAPI (Vector Labs) and sealed with nail polish to prevent drying.

L1 RNA was detected with the MS2-GFP labeling technique (66,76). HeLa-M cells were co-transfected with pAD3TE1 and pMS2-GFP as noted above. A nuclear-localization signal restricts GFP-MS2 chimera to the nucleus, where it associates with L1 RNA through MS2-binding sites present in the pAD3TE1 3′-UTR (66). Cytoplasmic GFP signals (white arrows, Figure 7) indicate the location of engineered L1 RNA after nuclear export.

All the primary and secondary antibodies were diluted in blocking buffer (3% goat serum, 0.1% Triton X-100 in 1× PBS). The following primary antibodies were used: rat anti-HA (clone 3F10, 1:100 dilution, Roche), mouse anti-EBV nuclear antigen (EBNA-1, 1:100 dilution, Abcam) and rabbit anti-Myc (1:200 dilution, Cell Signaling Technology). Highly cross-absorbed Alexa Fluor Dyes linked to goat IgG (H+L) secondary antibodies were used at 1:1000 dilution, including Alexa Fluor 488 goat anti-mouse, Alexa Fluor 568 goat anti-mouse, Alexa Fluor 568 goat anti-rat and Alexa Fluor 647 goat anti-rabbit (Molecular Probes). Notably, the entire slide was examined and representative images of each sample slide were captured using a Leica TCS-SP confocal microscope with fluorescent filters.

## RESULTS

### RNase L suppresses the retrotransposition of engineered L1 and IAP elements

We used previously established cell culture assays to determine whether the expression of an RNase L cDNA affects the retrotransposition of engineered human L1 and mouse IAP retrotransposons (Figure 1) (13,41,73). Briefly, HeLa-M cells, which are deficient in endogenous RNase L expression (71) (Figure 3C, Lane 1), were co-transfected with either an engineered retrotransposon [human L1 pJM101/L1.3 (20)] or mouse IAP [pDJ33/440N1*neo^TNF^* (13)] and either an empty vector (pFLAG-CMV-2) or a plasmid that expresses an amino-terminal FLAG-tagged version of RNase L [WT RNase L, a catalytically inactive RNase L mutant (R667A), or constitutively active (NΔ385) RNase L mutant (70)]. While the HeLa-M cells used in this study are deficient in RNase L, it should be noted that other types of HeLa cells (including ATCC CCL-2 and S3) express normal levels of endogenous RNase L (77,78). Both the L1 and IAP constructs contain a retrotransposition indicator cassette in their 3′ ends (Figure 1). The indicator cassette consists of either an antisense copy of a neomycin phosphotransferase gene (*mneoI*) or an enhanced green fluorescent protein-coding gene (*mEGFPI*) equipped with a heterologous promoter (Pr′) and polyadenylation signal (lollipop symbol, Figure 1A) (41,65,79). Notably, both the *mneoI* and *mEGFPI* indicator cassettes are disrupted by an intron [IVS2 of the γ-globin gene in the L1 constructs (41) and intron 2 of the murine tumor necrosis factor beta (TNF-β) gene in the IAP construct (80)] that is in the same transcriptional orientation as the L1 or IAP retrotransposon. This arrangement ensures that the reporter gene only will become activated and expressed if the retrotransposon RNA is reverse transcribed and integrated into genomic DNA (Figure 1B). The resultant numbers of G418-resistant foci or EGFP-positive cells serve as a read out of L1 or IAP retrotransposition efficiency (Figure 1C) (41,65).

**Figure 2.**
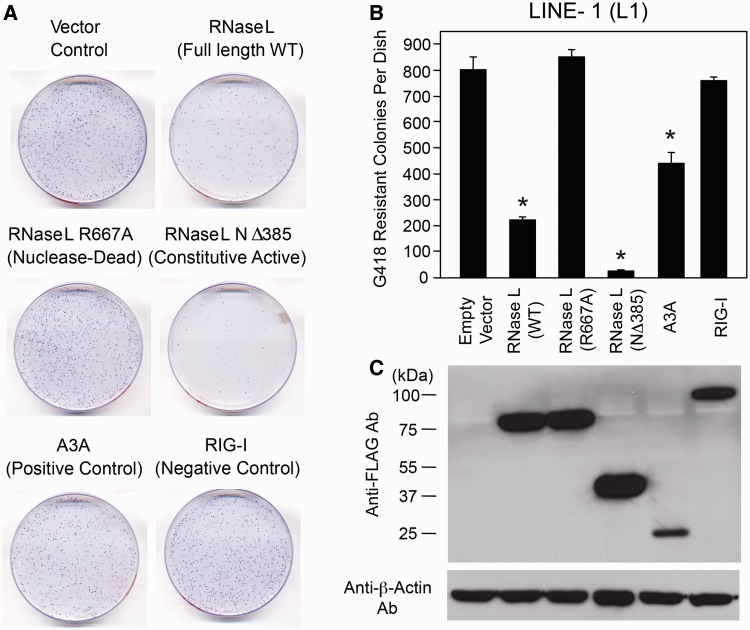
Inhibition of L1 retrotransposition by RNase L. (**A**) L1 Retrotransposition Assays: HeLa-M cells were co-transfected with pJM101/L1.3 and either an empty vector (pFLAG-CMV-2) or a plasmid that encodes amino-terminal FLAG-tagged versions of the following proteins: RNase L, A3A or RIG-I. The cells were subjected to selection for 10 days and G418-resistant foci were fixed and stained with crystal violet for visualization purposes. A representative tissue culture dish for each condition is shown. (**B**) Quantitation of the L1 Retrotransposition Assays: The X-axis depicts the co-transfected construct names. The Y-axis depicts the number of G418-resistant foci per cell culture dish. Data are shown as the mean ± standard deviation (SD) from a single experiment with three technical replicates. *P < 0.01 (when each test group was compared with empty vector control with Dunnett’s Multiple Comparison Test). The experiment was conducted four times (biological replicates) with similar results. (**C**) Protein expression analyses: The WT RNase L, catalytically inactive RNase L mutant (R667A), constitutively active (NΔ385) RNase L mutant, A3A and RIG-I proteins were detected from total cell lysates in western blots with anti-FLAG antibody 2 days after transfection. β-actin served as loading and transfer control. Size standards are indicated in kDa at the left of the gel.

**Figure 3.**
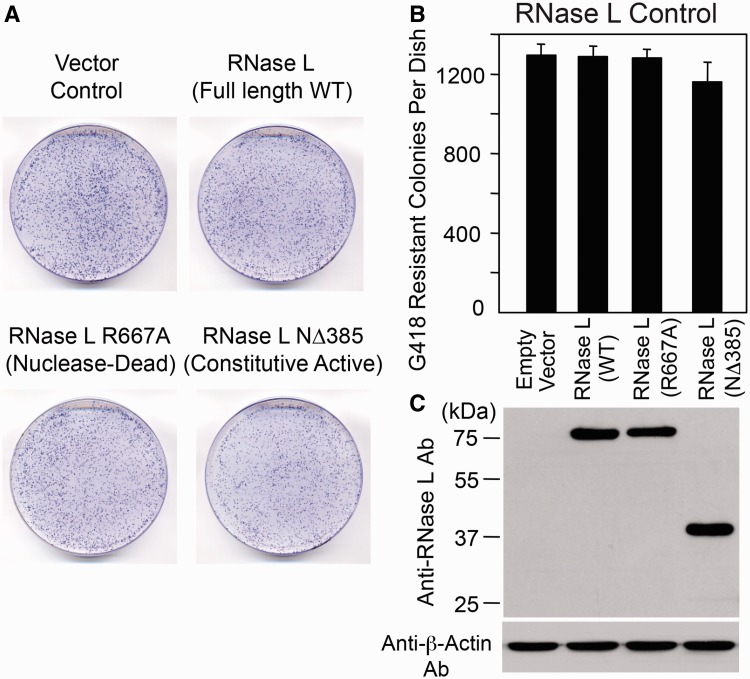
Human RNase L alone does not affect G418-resistant foci formation. (**A**) Results from the Assay: HeLa-M cells were co-transfected with pcDNA 3.0 (Gibco/Life Technologies/InVitrogen) and either an empty vector (pFLAG-CMV-2) or an amino-terminal FLAG-tagged RNase L expression plasmid. The cells were subjected to selection for 10 days and G418-resistant foci were fixed and stained with crystal violet for visualization purposes. A representative tissue culture dish for each condition is shown. (**B**) Quantitation of the Assays: The X-axis depicts construct names. The Y-axis depicts the number of G418-resistant foci per cell culture dish. Quantification was performed as outlined in the legend to Figure 2B. Data are shown as the mean ± standard deviation (SD) from a single experiment with three technical replicates. The experiment was conducted three times (biological replicates) with similar results. No statistically significant difference was found with one-way ANOVA and post hoc tests. (**C**) Protein expression analyses: The WT RNase L, catalytically inactive RNase L mutant (R667A) and constitutively active (NΔ385) RNase L mutant were detected from total cell lysates in western blots with anti-RNase L antibody 2 days after transfection. β-Actin served as loading and transfer control. Size standards are indicated in kDa at the left of the gel.

**Figure 4.**
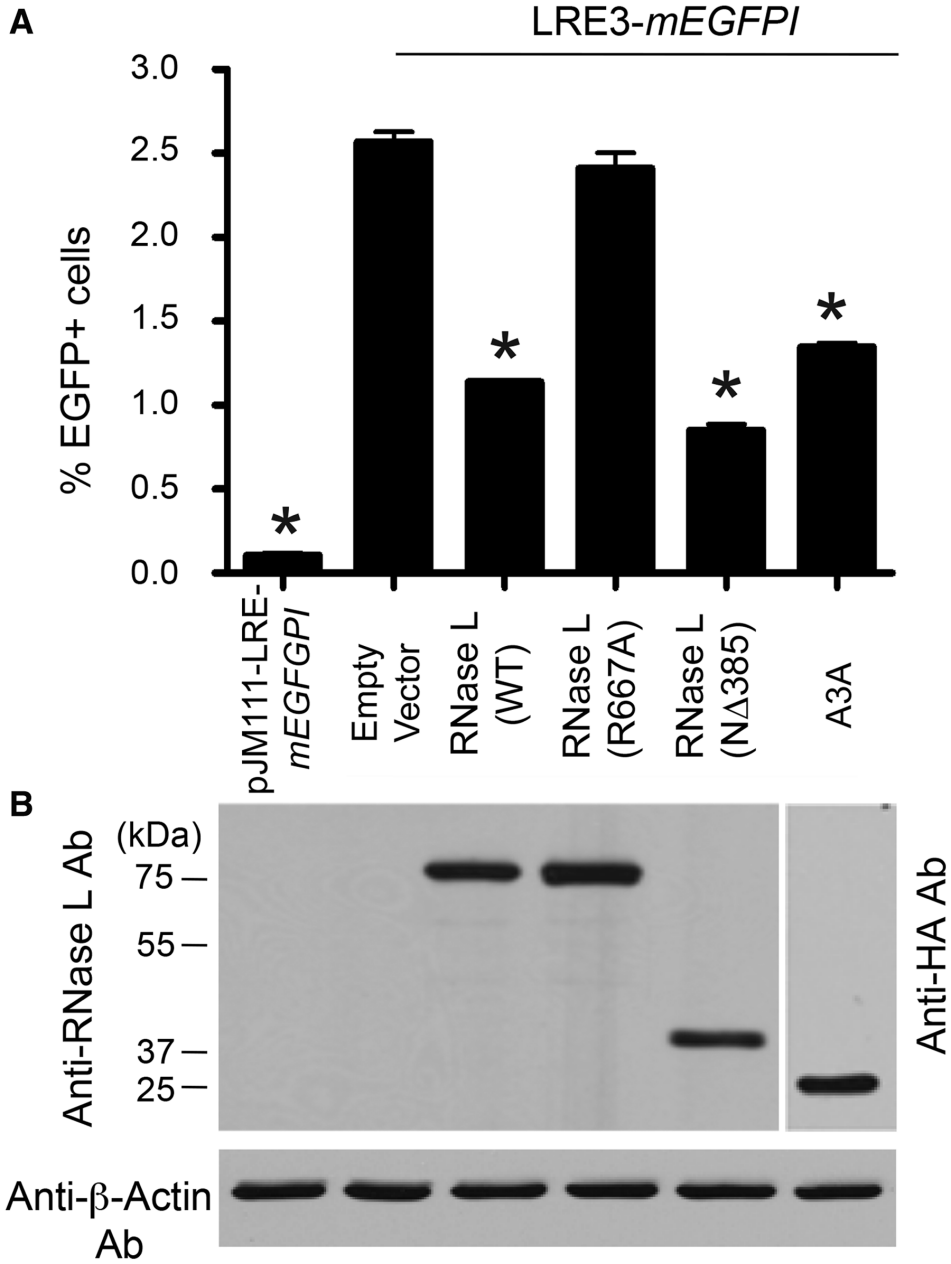
Expression of RNase L inhibits L1 retrotransposition in an *EGFP*-based retrotransposition assay. (**A**) Results from the assays: HeLa-M cells were co-transfected with an expression construct containing an active human L1 (pLRE3-*mEGFPI*) and an empty vector (pFLAG-CMV-2), a plasmid encoding an amino-terminal FLAG-tagged RNase L expression plasmid or an amino-terminal HA-tagged A3A expression plasmid. Experiments with a retrotransposition-defective L1 pJM111-LRE3-*mEGFPI* served as a negative control. The cells were subjected to puromycin selection for 4 days after transfection. Fluorescence Activated Cell Sorting (FACS) was then used to screen for EGFP-positive cells. The X-axis indicates the construct name. The Y-axis indicates the percentage of EGFP-positive cells. For each sample, 2 × 10^5^ cells were analyzed and the percentage of EGFP-positive cells was calculated with using the FlowJo software package. Data were analyzed with one-way ANOVA with post hoc tests and are shown as mean ± SD from a single experiment with three technical replicates. *P < 0.01 (Dunnett’s Multiple Comparison Test). The experiment was conducted four times (biological replicates) with similar results. (**B**) Protein expression analyses: The WT RNase L, catalytically inactive RNase L mutant (R667A), and constitutively active (NΔ385) RNase L mutants were detected in total cell lysates by western blot with anti-RNase L antibody 2 days after transfection. The A3A protein was detected using an anti-HA antibody. β-Actin served as loading and transfer control. Size standards are indicated in kDa at the left of the gel.

Retrotransposition assays revealed that expression of FLAG-tagged WT or constitutively active RNase L mutant (NΔ385) proteins (70) led to a reduction in L1 retrotransposition efficiency in the *mneoI*-based reporter assays (∼72 and ∼97%, respectively) (Figure 2A and B). In contrast, the FLAG-tagged catalytically inactive RNase L mutant (R667A) did not significantly inhibit L1 retrotransposition (Figure 2A and B). As a positive control, we demonstrated that the expression of a FLAG-tagged A3A protein reduced L1 retrotransposition by ∼50%. The lower level of A3A expression (Figure 2C) may lead to its reduced suppression of L1 retrotransposition when compared with previous studies (68,81). As a negative control, we demonstrated that the expression of FLAG-tagged RIG-I protein (82), a pathogen recognition receptor for viral RNA, had no significant effect on L1 retrotransposition. Western blot analyses confirmed that FLAG-tagged RNase L, A3A and RIG proteins were expressed in Hela-M cells (Figure 2C). Moreover, the expression of the RNase L proteins did not significantly impact cell viability (Figure 3). To corroborate the above findings, we next tested whether the expression of FLAG-tagged WT RNase L, constitutively active RNase L mutant (NΔ385) and catalytically inactive RNase L mutant (R667A) proteins could inhibit the mobility of an engineered human L1 that contains the *mEGFPI*-based retrotransposition cassette located in the 3′-UTR (Figure 1A; pLRE3-*mEGFPI*) (19,65). Once again, we found that both the WT and NΔ385 RNase L proteins inhibited L1 retrotransposition and that the catalytically inactive R667A RNase L mutant did not (Figure 4A and Supplementary Figure S1). The control plasmid (pJM111-LRE3-*mEGFPI*), which carries two missense mutations in ORF1p rendering it inactive, showed only background EGFP expression. Western blot analyses confirmed that the epitope-tagged RNase L and A3A proteins were expressed (Figure 4B). Notably, the decrease in retrotransposition efficiency (∼50%) caused by RNase L expression was less pronounced in the *EGFP*-based retrotransposition assays when compared with the *mneoI*-based retrotransposition assay. These differences may be due to the shorter time duration of the *mEGFPI*-based retrotransposition assays (6 days) when compared with *mneoI*-based (12 day) assays.

**Figure 5.**
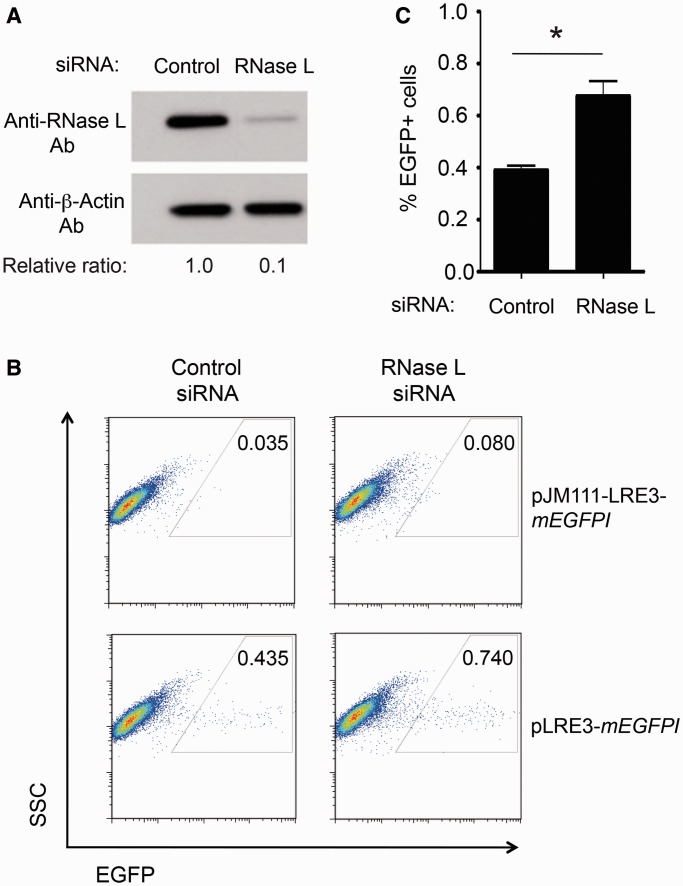
Depletion of endogenous RNase L increases L1 retrotransposition efficiency. (**A**) Knockdown of endogenous RNase L protein: Hey1b cells were transfected with control siRNA pools or RNase L siRNA pools. Western blotting using an anti-RNase L monoclonal antibody confirmed RNase L knockdown 48 h after siRNA transfection. β-Actin served as loading and transfer control. The band intensity was quantified with ImageJ software (83) and the relative ratio of RNase L to β-actin is shown. (**B**) Representative Retrotransposition Assay Results: Control siRNA (control) and siRNA-mediated RNase L depleted cells (RNase L) were transfected with either pLRE3-*mEGFPI* or pJM111-LRE3-*mEGFPI*. L1 retrotransposition was assayed as described in Figure 4. Representative FACS plots are shown, as is the conservative gating strategy used to detect EGFP-positive cells. (**C**) Quantitation of the Retrotransposition Assays: The X-axis indicates the control siRNA (control) or siRNA-mediated RNase L depleted cells (RNase L). The Y-axis indicates the percentage of EGFP-positive cells. For each sample, 2 × 10^5^ cells were analyzed and the percentage of EGFP-positive cells was calculated with using the FlowJo software package. The experiment was conducted four times (biological replicates) with similar results; representative data from one experiment are shown. Data are reported as the mean ± SD from three technical replicates of a single representative experiment. The asterisk indicates a *P* = 0.0079 and was calculated with two-tailed Student’s *t*-test.

To test whether endogenous RNase L restricts L1 retrotransposition, we used siRNA-based experiments to deplete RNase L in a human ovarian cancer cell line, Hey1b (Supplementary Figure S2). Hey1b cells express relatively high levels of RNase L typical of some human cancer cell lines, in contrast, RNase L is barely detectable in HeLa-M cells (71). Western blot analyses, using a monoclonal antibody that detects endogenous RNase L, revealed that cells transfected with an siRNA pool that targets RNase L exhibited an ∼90% reduction in endogenous RNase L protein levels when compared with cells transfected with a control siRNA pool (Figure 5A). Reduction of the endogenous RNase L protein level was evident 24 h after transfection and was maintained for ∼96 h (data not shown). Retrotransposition assays using pLRE3-*mEGFPI* (19,65) revealed that ∼0.4% of cells treated with control siRNA became EGFP-positive after 4 days of puromycin drug selection (Figure 5B and C and Supplementary Figure S2), which enriched for cells containing the L1 expression plasmid. In contrast, ∼0.75% of cells treated with siRNA against RNase L were EGFP-positive (Figure 5B and C). As expected, the retrotransposition-defective L1 (pJM111-LRE3-*mEGFPI*) only exhibited background EGFP expression levels regardless of RNase L depletion (Figure 5B).

**Figure 6.**
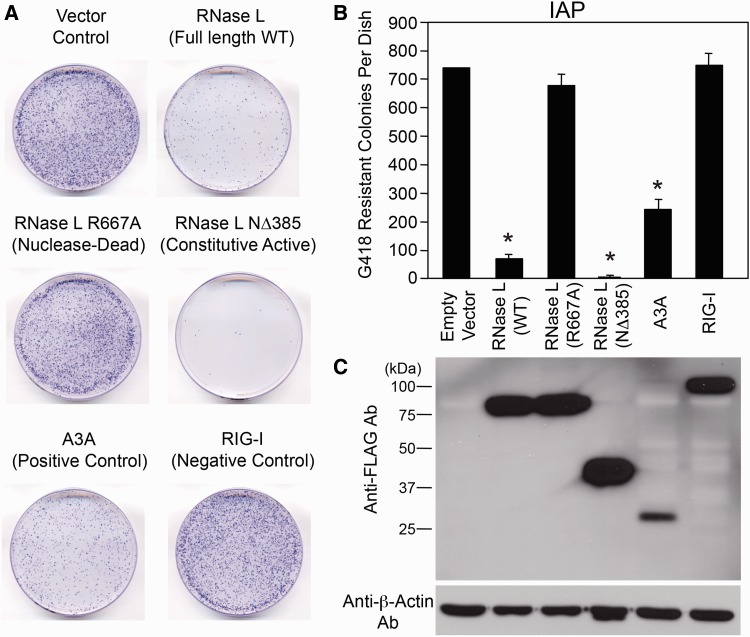
Inhibition of IAP retrotransposition by RNase L. (**A**) IAP Retrotransposition Assays: HeLa-M cells were co-transfected with a mouse IAP expression construct (pDJ33/440N1*neo^TNF^*) and either an empty vector (pFLAG-CMV-2) or an expression plasmid that encodes an amino-terminal FLAG-tagged version of the following proteins: WT RNase L, a catalytically inactive RNase L mutant (R667A), a constitutively active RNase L mutant (NΔ385), A3A or RIG-I. The cells were subject to selection for 10 days and G418-resistant foci were fixed and stained with crystal violet for visualization purposes. A representative tissue culture dish for each condition is shown. (**B**) Quantitation of the IAP Retrotransposition Assays: The X-axis depicts names of constructs co-transfected into cells with the IAP construct. The Y-axis depicts the number of G418-resistant foci per cell culture dish. Data are represented as the mean ± standard deviation (SD) from a single experiment with three technical replicates. *P < 0.01 (when each test group was compared with empty vector control with Dunnett’s Multiple Comparison Test). The error bar in the Empty Vector bar is too small to visualize. The experiment was conducted three times (biological replicates) with similar results. (**C**) Protein expression analyses: The WT RNase L, catalytically inactive RNase L mutant (R667A), constitutively active RNase L mutant (NΔ385), A3A and RIG-I proteins were detected in total cell lysates by western blotting with anti-FLAG antibody 2 days after transfection. β-Actin served as loading and transfer control. Size standards are indicated in kDa at the left of the gel.

We next determined if RNase L was able to repress the retrotransposition of an engineered mouse IAP element (Figure 1A, construct pDJ33/440N1*neo^TNF^*). Consistent with our L1 findings, expression of the WT and constitutively active RNase L proteins severely reduced IAP retrotransposition efficiency by ∼90% (Figure 6). The catalytically inactive RNase L (R667A) mutant did not significantly affect IAP retrotransposition. Again, controls indicated that the expression of A3A reduced IAP retrotransposition, whereas RIG-I expression did not significantly affect retrotransposition (Figure 6B). Together, the above data strongly suggest that RNase L is a potent inhibitor of engineered L1 and IAP retrotransposition and that this inhibition requires RNase L nuclease activity.

### RNase L reduces levels of L1 RNA

**Figure 7.**
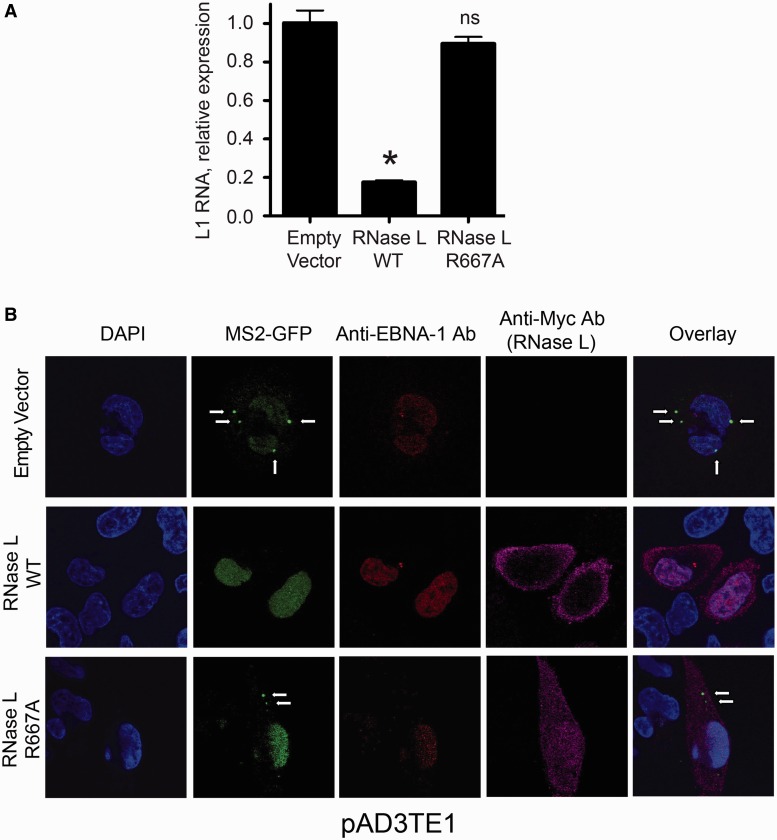
RNase L reduces L1 RNA accumulation in cells. (**A**) Results of qRT-PCR experiments: HeLa-M cells were co-transfected with pAD2TE1 and an empty vector (pFLAG-CMV-2) or an amino-terminal FLAG-tagged RNase L expression plasmid. L1 RNA levels were determined 48 h after transfection using the Sybr Green method (84). The X-axis indicates the RNase L co-transfected samples. The Y-axis indicates the relative expression level of L1 RNA from the transfected construct. The L1 RNA amounts were normalized with hygromycin mRNA levels (see ‘Materials and Methods’ section for detailed PCR strategy). Data are represented as the mean ± SD from three technical replicates of a single representative experiment. *P < 0.01 (Dunnett’s Multiple Comparison Test). The experiment was conducted three times (biological replicates) with similar results. ns, not significant. (**B**) Immunofluorescent Confocal Microscopy Studies: HeLa-M cells were co-transfected with pAD3TE1, a plasmid expressing a nuclear localized MS2-GFP fusion protein, and an empty vector (pcDNA 3.0) or an amino-terminal Myc-tagged RNase L expression plasmid. Immunofluorescent confocal microscopy demonstrated L1 RNA accumulation in cytoplasmic foci by exploiting the 24 MS2 binding sites in pAD3TE1 L1 RNA. The top labels indicate DAPI, MS2-GFP or the antibodies used to detect the EBNA-1 and RNase L proteins. The labels on the left side of the figure indicate the empty vector or RNase L constructs that were co-transfected into cells. The rightmost column indicates the overlay staining. The white arrows indicate L1 cytoplasmic foci containing L1 RNA. For each condition, either two or three slides were examined per experiment. About 200 cells were examined per slide and representative images were captured. The experiment was conducted three times (biological replicates) with similar results.

Since RNase L is a ribonuclease, we next asked if its expression affected steady state L1 mRNA levels in transfected cells. To accomplish this goal, we designed primers that would amplify a 91-bp fragment that spans the junction of the ORF2/TAP-tag coding region in a transfected L1 expression construct (pAD2TE1) (66). Notably, these primers should specifically amplify L1 mRNA derived from pAD2TE1 and should not amplify endogenous L1 mRNAs, which lack the TAP epitope-tag at the end of the ORF2 coding sequence. Primers capable of amplifying mRNA from the hygromycin phosphotransferase gene (HYG) present on the pCEP4 L1 expression plasmid backbone served as an internal/normalization control. [The polymerase chain reaction (PCR) strategy is illustrated in Figure 1A, see PCR primers beneath the map of pAD2TE1.] Quantitative reverse transcriptase PCR (RT-PCR) analysis revealed that the expression of WT RNase L reduced L1 mRNA levels by ∼80% (Figure 7A). However, expression of the catalytically inactive R667A RNase L mutant failed to significantly reduce L1 mRNA levels (Figure 7A). In a separate control experiment, we demonstrated that co-transfection of WT RNase L did not affect HYG mRNA levels expressed from pIREShyg (Clontech) (Supplementary Figure S3). These data suggest that RNase L preferentially targets L1 mRNA for degradation, and that its nuclease activity is required for the decrease in L1 mRNA levels.

To validate our quantitative RT-PCR (qRT-PCR) findings, we analyzed the effect of RNase L on the accumulation of L1 mRNA in the cytoplasm of cells. To visualize the L1 mRNA, we took advantage of a previously described construct, pAD3TE1, which encodes an L1 element that contains 24 copies of MS2 RNA binding element in its 3′-UTR (Figure 1A) (66). The co-expression of pAD3TE1 and a plasmid encoding a nuclear localized MS2-GFP protein would allow the MS2-GFP protein to bind the MS2 RNA sequences in pAD3TE1 mRNA, allowing the indirect visualization of the L1 mRNA via immunofluorescent confocal microscopy (66). We observed punctate L1 cytoplasmic foci in cells co-transfected with pAD3TE1, the MS2-GFP protein expression construct and either the empty vector pcDNA 3.0 or the inactive RNase L R667A mutant (Figure 7B). In contrast, we did not observe punctate L1 cytoplasmic foci in cells co-transfected with pAD3TE1, the MS2-GFP protein expression construct and the WT RNase L expression construct (∼200 cells were examined per slide and representative images were captured) (Figure 7B). As an additional control for this experiment, we determined that EBNA-1, which is present on the backbone of the pAD3TE1 expression vector, was still expressed in the presence of WT RNase L. Together, these data indicate a ribonuclease-dependent effect of RNase L on L1 RNA levels and likely explain, in part, how RNase L adversely affects L1 retrotransposition.

### Expression of RNase L leads to a decrease in L1 protein expression

During viral infections, the OAS-RNase L system degrades certain viral and cellular mRNAs, thereby preventing protein synthesis [reviewed in (56)]. Thus, we performed western blot analyses to determine if the observed reduction in L1 mRNA correlated with a reduction in the accumulation of the L1-encoded proteins. To accomplish this goal, we co-transfected HeLa-M cells with pAD2TE1 (66) and either an empty vector (pFLAG-CMV-2) or a FLAG-tagged RNase L expression construct (WT, the constitutively active NΔ385 RNase L mutant or the catalytically inactive R667A RNase L mutant). The transfected cells then were subjected to selection in hygromycin B containing medium. The total cell lysates and L1 RNP preparations then were subjected to western blot analyses (see ‘Materials and Methods’ section).

**Figure 8.**
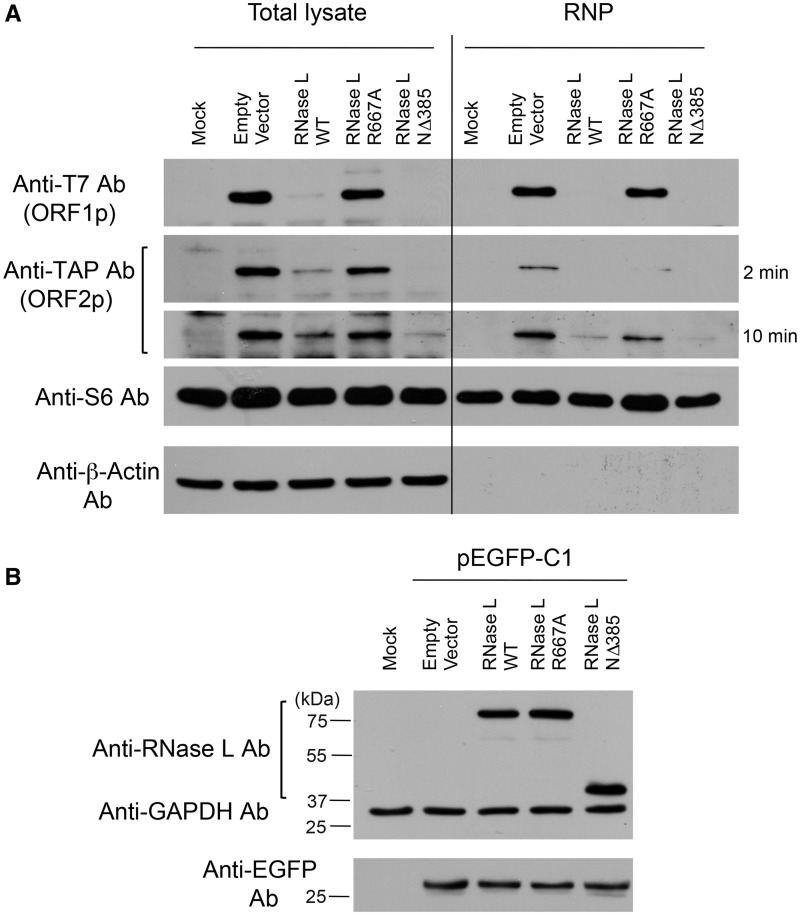
Expression of RNase L reduces L1 protein expression. (**A**) L1 protein expression: HeLa-M cells were co-transfected with pAD2TE1 and either an empty vector (pFLAG-CMV-2) or a plasmid that encodes an amino-terminal FLAG-tagged RNase L expression plasmid. Two days after transfection, cells were selected with hygromycin containing medium for an additional 4 days when total cell lysates and L1 RNPs were prepared. Western blotting, using anti-T7 and anti-TAP antibodies, was used to detect ORF1p and ORF2p, respectively. Shown are two exposures of the ORF2p anti-TAP western blot. Endogenous ribosomal S6 protein was used as the loading/transfer control. β-Actin detection discriminated the total cell lysate (left side of panel) from the L1 RNP fractions (right side of panel). The experiments were repeated twice (biological replicates) with similar results. Shown are data from one representative experiment. (**B**) RNase L does not inhibit exogenous EGFP protein expression: HeLa-M cells were co-transfected with pEGFP-C1 and either an empty vector (pFLAG-CMV-2) or a plasmid that encodes an amino-terminal FLAG-tagged RNase L expression plasmid. Total cell lysates were harvested and the expression of RNase L and GFP was detected in western blot experiments using anti-RNase L and anti-GFP antibodies at 48 h after transfection. GAPDH served as a loading and transfer control.

An anti-T7 antibody detected the ∼40 kDa ORF1p in both total cell lysates and RNP fractions derived from cells co-transfected with pAD2TE1 and either an empty vector (pFLAG-CMV-2) or the catalytically inactive RNase L (R667A) mutant (Figure 8A). Similarly, an anti-TAP antibody detected the ∼170 kDa ORF2p in both total cell lysates and RNP fractions. In contrast, ORF1p and ORF2p were markedly reduced in cells transfected with pAD2TE1 and either the WT RNase L or constitutively active NΔ385 RNase L mutant. Notably, RNase L did not affect the level of endogenous ribosomal S6 protein in total cell lysates and RNP fractions. We also observed an RNase L-dependent reduction of ORF1p in cells transfected with pDK500 (a T7-*gene 10* epitope-tagged ORF1p expression construct), as well as a reduction in ORF2p in cells transfected with pAD500 (a TAP epitope-tagged ORF2p expression construct) (Supplementary Figures S4 and S5, respectively). Control assays again demonstrated that the WT and mutant FLAG-tagged RNase L proteins are expressed at similar levels in total cell lysates 2 days after transfection (Figure 8B). Importantly, we did not observe an RNase L-dependent reduction in HeLa-M cells co-transfected with pEGFP-C1, suggesting that RNase L may preferentially target L1 RNA, thereby adversely affecting the expression of the L1 proteins (Figure 8B).

### Expression of RNase L prevents the formation of L1 cytoplasmic foci

**Figure 9.**
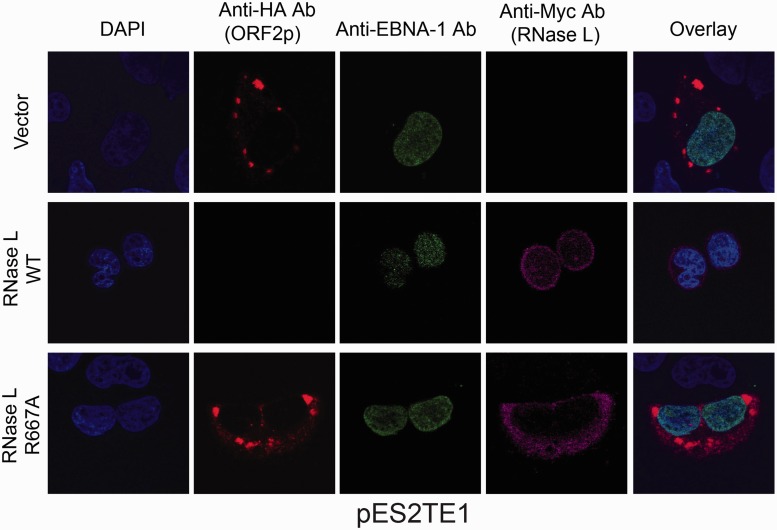
Expression of RNase L blocks L1 RNP formation. HeLa-M cells were co-transfected with pES2TE1 and either an empty vector (pcDNA 3.0) or a plasmid that encodes an amino-terminal Myc-tagged RNase L expression plasmid. Immunofluorescent confocal microscopy was used to examine L1 ORF2p accumulation in cytoplasmic foci by exploiting the FLAG-HA epitope-tag in pES2TE1 48 h after transfection. The top labels indicate the antibodies used to detect the indicated proteins: anti-HA-ORF2p, red; anti-EBNA-1, green; anti-Myc RNase L, magenta. The labels on the left side of the figure indicate the empty vector or RNase L constructs that were co-transfected into cells. The rightmost column indicates the merged overlay staining. L1 ORF2p formed discrete cytoplasmic punctate localization in co-transfection experiments performed with the empty vector and RNase L catalytically inactive mutant (R667A), but not with WT RNase L. For each condition, either two or three slides were examined per experiment. About 200 cells were examined per slide and representative images were captured. The experiment was conducted three times (biological replicates) with similar results.

Previous immunofluorescence studies demonstrated that the L1-encoded proteins and L1 RNA begin to appear as discrete cytoplasmic foci that often associate with stress granules and/or processing bodies (P-bodies) at 48 h posttransfection (66,85). Because the expression of RNase L adversely affected L1 mRNA levels and, in turn, ORF1p and ORF2p protein accumulation, we next examined whether RNase L expression adversely affects L1 cytoplasmic foci accumulation. To accomplish this goal, we co-transfected HeLa-M cells with an L1 expression vector (pES2TE1) (66) including an HA-tagged ORF2p, and an empty vector (pcDNA 3.0), a plasmid expressing the amino-terminal Myc-tagged WT RNase L or a catalytically inactive R667A RNase L mutant expression construct. Consistent with the findings reported above, immunofluorescent confocal microscopy revealed the presence of ORF2p cytoplasmic foci in the presence of the empty vector and the catalytically inactive R667A RNase L mutant expression construct (Figure 9). In contrast, L1 ORF2p foci were not observed on co-expression of WT RNase L.

Additional control experiments revealed expression of the EBNA-1 protein from the pCEP4 plasmid backbone in either the presence or absence of WT RNase L (Figure 9, green signal), confirming that the plasmids were successfully transfected into cells. Moreover, we confirmed that both the WT and R667A RNase L mutant were expressed in Hela-M cells, and exhibited a diffuse cytoplasmic localization (Figure 9, magenta signal). Although RNase L previously was reported to associate with stress granules after viral infection (86), we did not detect co-localization of RNase L and ORF2p, possibly because RNase L degraded L1 RNA, thereby inhibiting L1 protein expression and RNP formation. Together, the above data are not inconsistent with the conclusion that RNase L may preferentially target L1 transcripts for degradation.

## DISCUSSION

### Mechanism of L1 restriction by RNase L

Our findings strongly suggest that the relatively general antiviral pathway mediated by RNase L may also restrict certain non-LTR and LTR retrotransposons. The available data indicate that transient expression of RNase L inhibits both L1 and IAP retrotransposition (Figures 2, 4 and 6). Conversely, the siRNA-mediated knockdown of endogenous RNase L increased L1 retrotransposition by ∼87% (Figure 5). Regarding our IAP results, while there are limitations in drawing conclusions from cross species transfection experiments, our findings suggest a relatively general role for RNase L in restricting retrotransposons that have different integration mechanisms. IAP elements in mice are the result of transmission of the viral progenitor IAPE (87), and therefore, inhibition of IAP transposition by RNase L is not inconsistent with its antiviral function.

L1 and IAP retrotransposition are not suppressed in the presence of an RNase L containing a single amino acid substitution (R667A), which abrogates its enzymatic activity. These data indicate that a principal mechanism by which RNase L inhibits L1 and IAP retrotransposition likely involves the posttranscriptional cleavage of retrotransposon RNA. In addition, it is unknown if R667A also affects RNA binding, but if it does it could explain why the catalytically inactive mutant cannot be co-localized with L1 RNA. The expression of WT RNase L, as well as a constitutively active RNase L mutant (NΔ385), led to a reduction in L1 RNA levels; this reduction, in turn, led to a decrease in both ORF1p and ORF2p expression (Figures 7, 8 and 9). In contrast, RNase L expression had little effect on HYG mRNA, which also is expressed from the L1 expression construct (pCEP4) backbone (Figure 7). These findings and a lack of ribosomal RNA cleavage products (data not shown) suggest that RNase L preferentially targets L1 RNA for degradation.

The specificity of RNase L has been previously studied (88,89). RNase L cleaves single-strand RNA, predominantly after UpAp and UpUp dinucleotide sequences (88,89). However, the structural context of the RNA substrate greatly influences the choice of cleavage sites (90). Certain viral and cellular single-strand RNAs are subject to degradation. For example, ribosomal RNA present in intact ribosomes can be cleaved by RNase L producing a characteristic pattern of discrete products in some IFN-treated and virus-infected cells (91,92). The molecular mechanism by which RNase L targets L1 RNA requires further study. We hypothesize that double-stranded structures (e.g. stem loops) within L1 RNA may activate OAS to produce microdomains of 2-5A (an activator of RNase L) from ATP. Alternatively, RNAs produced from the L1 sense and antisense promoters (SP and ASP, respectively) (46) may hybridize to form a region of dsRNA that activates OAS (46,93). This proposed localized activation of OAS, and RNase L may then result in the targeted degradation of L1 RNA.

The above model has precedent. For example, it previously was hypothesized that partially double-stranded RNAs, such as replicative intermediates from certain picornaviruses including encephalomyocarditis virus, could lead to localized OAS-RNase L activation (94,95). RNAs linked to double-strand RNA were preferentially degraded by RNase L when compared with single-stranded RNAs lacking a double-strand segment (94). By analogy, there is evidence for another localized activation model involving production of a different small molecule second messenger, cyclic adenosine monophosphate (cAMP). The distribution in rat neonatal cardiac myocytes of enzymes that synthesize and degrade cAMP produce microdomains of cAMP that specifically activate a subset of localized protein kinase A molecules (96). Thus, in principle, 2-5A microdomains would form near the sites of OAS complexed with its RNA activators. Within these 2-5A microdomains, RNase L would become active causing cleavage of the RNA stimulators of OAS, in this case L1 RNA. This model could be relevant to both retrotransposition and viral infections and warrants further investigation. Finally, it is noteworthy that RNase L may not be the only host protein that regulates L1 retrotransposition by nucleic acid degradation. For example, it is hypothesized that the 3′-5′ exonuclease Trex1 inhibits L1 retrotransposition by degrading L1 cDNA intermediates (48).

Notably, we did not observe co-localization of RNase L with L1 cytoplasmic foci (Figures 7 and 8). There are a number of possible explanations for this result. First, the degradation of L1 RNA by WT or constitutively active RNase L would be predicted to inhibit the formation of L1 RNPs, thereby hampering the visualization of co-localized foci. Second, the inability to observe co-localization of L1 cytoplasmic foci with the catalytically inactive R667A RNase L mutant could reflect the transient nature of the association of RNase L with its RNA substrate (97). Similarly, the absence of RNase L in the interactome from isolated L1 ORF1 protein and its RNPs is not inconsistent with a transient interaction between in L1 RNA and RNase L (54). Lastly, it remains possible that the ectopic expression of RNase L leads to an artifactual degradation of L1 RNA. However, this scenario is unlikely because RNase L did not inhibit HYG mRNA production or EGFP protein production, and siRNA-mediated depletion of RNase L from Hey1b cell led to a modest increase in L1 retrotransposition (Figures 5, 7A and 8B).

### Implications for RNase L in the control of retrotransposons

RNase L was previously suggested to be involved in prostate carcinogenesis after being mapped to the hereditary prostate cancer 1 locus (98). Mutations in RNase L discovered from linkage analysis include two protein inactivating mutations (Δ157-164X and E265X), a mutation that abrogates translation (M1I) and a missense variant 1385G→A (R462Q) that reduce RNase L activity by 3-fold (99). The connection between RNase L and prostate cancer was further expanded to other types of cancer. Genetic variations in *RNASEL* have been identified in cancers of head and neck, uterus, cervix and breast (100). They are also associated with disease aggressiveness and metastasis in familial pancreatic cancer (101) and with age of onset of hereditary nonpolyposis colon cancer (102).

Studies from our group and others found that loss-of-function mutations in *RNASEL* potentially contributed to cancer development by dysregulating apoptosis of cancer cells (71,103–105). However, inconsistent findings on the same *RNASEL* mutations among studies, some that show an association with cancer and others that do not, suggest that RNase L might act as a modifier of disease progression with possible interactions between environmental factors and genetics [reviewed in (99,106)]. In this regard, it would be interesting to see if a loss of RNase L activity correlates with an increase in L1 retrotransposition activity in certain tumors. Recent studies have shown somatic L1 retrotransposition activity in a subset of colorectal, liver and lung tumors (107–110).

In conclusion, we have identified a potential restriction mechanism for retrotransposition involving the antiviral protein RNase L. These findings emphasize the complex and dynamic interplay between retrotransposons and the cell. Our data provide evidence that RNase L inhibits L1 RNA accumulation and the subsequent formation of L1 RNPs, thereby impairing the completion of the L1 retrotransposition cycle. By inhibiting L1 retrotransposition in somatic cells, RNase L might contribute to the maintenance of genomic stability.

## SUPPLEMENTARY DATA

Supplementary Data are available at NAR Online.

## FUNDING

National Institutes of Health, National Cancer Institute [CA044059]; the Maltz Family Foundation; the Mal and Lea Bank Chair fund (to R.H.S.); the National Institutes of Health [GM060518 to laboratory of J.V.M., who is an Investigator of the Howard Hughes Medical Institute (in part)]; International postdoctoral fellowship from the Fondation pour la Recherche Medicale (FRM) (to A.J.D. in part); NIH Training Grant [T32-GM007315 to J.B.M.] (in part). Funding for open access charge: NIH, National Cancer Institute [CA044059].

*Conflict of interest statement*. J.V.M. is listed as an inventor on the following patent, “Compositions and methods of use of human retrotransposons. Application No. 60/006,831; Patent number 6,150,160; Issued November 21, 2000.” He discloses this fact voluntarily. His involvement with the patent does not influence the results of this study.

## Supplementary Material

Supplementary Data

## References

[1] Lander ES, Linton LM, Birren B, Nusbaum C, Zody MC, Baldwin J, Devon K, Dewar K, Doyle M, FitzHugh W (2001). Initial sequencing and analysis of the human genome. Nature.

[2] Waterston RH, Lindblad-Toh K, Birney E, Rogers J, Abril JF, Agarwal P, Agarwala R, Ainscough R, Alexandersson M, An P (2002). Initial sequencing and comparative analysis of the mouse genome. Nature.

[3] Craig NL, Craig R, Gellert M, Lambowitz A (2002). Mobile DNA II.

[4] McClintock B (1950). The origin and behavior of mutable loci in maize. Proc. Natl Acad. Sci. USA.

[5] Pace JK, Feschotte C (2007). The evolutionary history of human DNA transposons: evidence for intense activity in the primate lineage. Genome Res..

[6] Mitra R, Li X, Kapusta A, Mayhew D, Mitra RD, Feschotte C, Craig NL (2013). Functional characterization of piggyBat from the bat *Myotis lucifugus* unveils an active mammalian DNA transposon. Proc. Natl Acad. Sci. USA.

[7] Levin HL, Moran JV (2011). Dynamic interactions between transposable elements and their hosts. Nat. Rev. Genet..

[8] Stoye JP (2012). Studies of endogenous retroviruses reveal a continuing evolutionary saga. Nat. Rev. Microbiol..

[9] Stocking C, Kozak CA (2008). Murine endogenous retroviruses. Cell. Mol. Life Sci..

[10] Beck CR, Garcia-Perez JL, Badge RM, Moran JV (2011). LINE-1 elements in structural variation and disease. Ann. Rev. Genomics Hum. Genet..

[11] Bannert N, Kurth R (2006). The evolutionary dynamics of human endogenous retroviral families. Ann. Rev. Genomics Hum. Genet..

[12] Bannert N, Kurth R (2004). Retroelements and the human genome: new perspectives on an old relation. Proc. Natl Acad. Sci. USA.

[13] Dewannieux M, Dupressoir A, Harper F, Pierron G, Heidmann T (2004). Identification of autonomous IAP LTR retrotransposons mobile in mammalian cells. Nat. Genet..

[14] Ribet D, Dewannieux M, Heidmann T (2004). An active murine transposon family pair: retrotransposition of “master” MusD copies and ETn trans-mobilization. Genome Res..

[15] Dewannieux M, Esnault C, Heidmann T (2003). LINE-mediated retrotransposition of marked Alu sequences. Nat. Genet..

[16] Hancks DC, Goodier JL, Mandal PK, Cheung LE, Kazazian HH (2011). Retrotransposition of marked SVA elements by human L1s in cultured cells. Hum. Mol. Genet..

[17] Raiz J, Damert A, Chira S, Held U, Klawitter S, Hamdorf M, Lower J, Stratling WH, Lower R, Schumann GG (2012). The non-autonomous retrotransposon SVA is trans-mobilized by the human LINE-1 protein machinery. Nucleic Acids Res..

[18] Hancks DC, Kazazian HH (2012). Active human retrotransposons: variation and disease. Curr. Opin. Genet. Dev..

[19] Brouha B, Schustak J, Badge RM, Lutz-Prigge S, Farley AH, Moran JV, Kazazian HH (2003). Hot L1s account for the bulk of retrotransposition in the human population. Proc. Natl Acad. Sci. USA.

[20] Sassaman DM, Dombroski BA, Moran JV, Kimberland ML, Naas TP, DeBerardinis RJ, Gabriel A, Swergold GD, Kazazian HH (1997). Many human L1 elements are capable of retrotransposition. Nat. Genet..

[21] Scott AF, Schmeckpeper BJ, Abdelrazik M, Comey CT, O'Hara B, Rossiter JP, Cooley T, Heath P, Smith KD, Margolet L (1987). Origin of the human L1 elements: proposed progenitor genes deduced from a consensus DNA sequence. Genomics.

[22] Dombroski BA, Mathias SL, Nanthakumar E, Scott AF, Kazazian HH (1991). Isolation of an active human transposable element. Science.

[23] Swergold GD (1990). Identification, characterization, and cell specificity of a human LINE-1 promoter. Mol. Cell. Biol..

[24] Minakami R, Kurose K, Etoh K, Furuhata Y, Hattori M, Sakaki Y (1992). Identification of an internal cis-element essential for the human L1 transcription and a nuclear factor(s) binding to the element. Nucleic Acids Res..

[25] Becker KG, Swergold GD, Ozato K, Thayer RE (1993). Binding of the ubiquitous nuclear transcription factor YY1 to a cis regulatory sequence in the human LINE-1 transposable element. Hum. Mol. Genet..

[26] Tchenio T, Casella JF, Heidmann T (2000). Members of the SRY family regulate the human LINE retrotransposons. Nucleic Acids Res..

[27] Athanikar JN, Badge RM, Moran JV (2004). A YY1-binding site is required for accurate human LINE-1 transcription initiation. Nucleic Acids Res..

[28] Speek M (2001). Antisense promoter of human L1 retrotransposon drives transcription of adjacent cellular genes. Mol. Cell. Biol..

[29] Holmes SE, Singer MF, Swergold GD (1992). Studies on p40, the leucine zipper motif-containing protein encoded by the first open reading frame of an active human LINE-1 transposable element. J. Biol. Chem..

[30] Hohjoh H, Singer MF (1996). Cytoplasmic ribonucleoprotein complexes containing human LINE-1 protein and RNA. EMBO J..

[31] Martin SL, Bushman FD (2001). Nucleic acid chaperone activity of the ORF1 protein from the mouse LINE-1 retrotransposon. Mol. Cell. Biol..

[32] Feng Q, Moran JV, Kazazian HH, Boeke JD (1996). Human L1 retrotransposon encodes a conserved endonuclease required for retrotransposition. Cell.

[33] Mathias SL, Scott AF, Kazazian HH, Boeke JD, Gabriel A (1991). Reverse transcriptase encoded by a human transposable element. Science.

[34] Esnault C, Maestre J, Heidmann T (2000). Human LINE retrotransposons generate processed pseudogenes. Nat. Genet..

[35] Wei W, Gilbert N, Ooi SL, Lawler JF, Ostertag EM, Kazazian HH, Boeke JD, Moran JV (2001). Human L1 retrotransposition: cis preference versus trans complementation. Mol. Cell. Biol..

[36] Kulpa DA, Moran JV (2006). Cis-preferential LINE-1 reverse transcriptase activity in ribonucleoprotein particles. Nat. Struct. Mol. Biol..

[37] Martin SL (1991). Ribonucleoprotein particles with LINE-1 RNA in mouse embryonal carcinoma cells. Mol. Cell. Biol..

[38] Kulpa DA, Moran JV (2005). Ribonucleoprotein particle formation is necessary but not sufficient for LINE-1 retrotransposition. Hum. Mol. Genet..

[39] Cost GJ, Feng Q, Jacquier A, Boeke JD (2002). Human L1 element target-primed reverse transcription *in vitro*. EMBO J..

[40] Luan DD, Korman MH, Jakubczak JL, Eickbush TH (1993). Reverse transcription of R2Bm RNA is primed by a nick at the chromosomal target site: a mechanism for non-LTR retrotransposition. Cell.

[41] Moran JV, Holmes SE, Naas TP, DeBerardinis RJ, Boeke JD, Kazazian HH (1996). High frequency retrotransposition in cultured mammalian cells. Cell.

[42] Belgnaoui SM, Gosden RG, Semmes OJ, Haoudi A (2006). Human LINE-1 retrotransposon induces DNA damage and apoptosis in cancer cells. Cancer Cell Int..

[43] Gasior SL, Wakeman TP, Xu B, Deininger PL (2006). The human LINE-1 retrotransposon creates DNA double-strand breaks. J. Mol. Biol..

[44] Siomi MC, Sato K, Pezic D, Aravin AA (2011). PIWI-interacting small RNAs: the vanguard of genome defence. Nat. Rev. Mol. Cell Biol..

[45] Ghildiyal M, Zamore PD (2009). Small silencing RNAs: an expanding universe. Nat. Rev. Genet..

[46] Yang N, Kazazian HH (2006). L1 retrotransposition is suppressed by endogenously encoded small interfering RNAs in human cultured cells. Nat. Struct. Mol. Biol..

[47] Chiu YL, Greene WC (2009). APOBEC3G: an intracellular centurion. Philos. Trans. R. Soc. Lond. B Biol. Sci..

[48] Stetson DB, Ko JS, Heidmann T, Medzhitov R (2008). Trex1 prevents cell-intrinsic initiation of autoimmunity. Cell.

[49] Goodier JL, Cheung LE, Kazazian HH (2012). MOV10 RNA helicase is a potent inhibitor of retrotransposition in cells. PLoS Genet..

[50] Arjan-Odedra S, Swanson CM, Sherer NM, Wolinsky SM, Malim MH (2012). Endogenous MOV10 inhibits the retrotransposition of endogenous retroelements but not the replication of exogenous retroviruses. Retrovirology.

[51] Li X, Zhang J, Jia R, Cheng V, Xu X, Qiao W, Guo F, Liang C, Cen S (2013). The MOV10 helicase inhibits LINE-1 mobility. J. Biol. Chem..

[52] Coufal NG, Garcia-Perez JL, Peng GE, Marchetto MC, Muotri AR, Mu Y, Carson CT, Macia A, Moran JV, Gage FH (2011). Ataxia telangiectasia mutated (ATM) modulates long interspersed element-1 (L1) retrotransposition in human neural stem cells. Proc. Natl Acad. Sci. USA.

[53] Peddigari S, Li PW, Rabe JL, Martin SL (2013). hnRNPL and nucleolin bind LINE-1 RNA and function as host factors to modulate retrotransposition. Nucleic Acids Res..

[54] Goodier JL, Cheung LE, Kazazian HH (2013). Mapping the LINE1 ORF1 protein interactome reveals associated inhibitors of human retrotransposition. Nucleic Acids Res..

[55] Dai L, Taylor MS, O'Donnell KA, Boeke JD (2012). Poly(A) binding protein C1 is essential for efficient L1 retrotransposition and affects L1 RNP formation. Mol. Cell. Biol..

[56] Silverman RH (2007). Viral encounters with 2′,5′-oligoadenylate synthetase and RNase L during the interferon antiviral response. J. Virol..

[57] Zhao L, Birdwell LD, Wu A, Elliott R, Rose KM, Phillips JM, Li Y, Grinspan J, Silverman RH, Weiss SR (2013). Cell-type-specific activation of the oligoadenylate synthetase-RNase L pathway by a murine coronavirus. J. Virol..

[58] Kerr IM, Brown RE (1978). pppA2′p5′A2′p5′A: an inhibitor of protein synthesis synthesized with an enzyme fraction from interferon-treated cells. Proc. Natl Acad. Sci. USA.

[59] Dong B, Silverman RH (1995). 2-5A-dependent RNase molecules dimerize during activation by 2-5A. J. Biol. Chem..

[60] Malathi K, Dong B, Gale M, Silverman RH (2007). Small self-RNA generated by RNase L amplifies antiviral innate immunity. Nature.

[61] Zhou A, Paranjape J, Brown TL, Nie H, Naik S, Dong B, Chang A, Trapp B, Fairchild R, Colmenares C (1997). Interferon action and apoptosis are defective in mice devoid of 2′,5′-oligoadenylate-dependent RNase L. EMBO J..

[62] Castelli JC, Hassel BA, Maran A, Paranjape J, Hewitt JA, Li XL, Hsu YT, Silverman RH, Youle RJ (1998). The role of 2′-5′ oligoadenylate-activated ribonuclease L in apoptosis. Cell Death Differ..

[63] Castelli JC, Hassel BA, Wood KA, Li XL, Amemiya K, Dalakas MC, Torrence PF, Youle RJ (1997). A study of the interferon antiviral mechanism: apoptosis activation by the 2-5A system. J. Exp. Med..

[64] Dombroski BA, Scott AF, Kazazian HH (1993). Two additional potential retrotransposons isolated from a human L1 subfamily that contains an active retrotransposable element. Proc. Natl Acad. Sci. USA.

[65] Ostertag EM, Prak ET, DeBerardinis RJ, Moran JV, Kazazian HH (2000). Determination of L1 retrotransposition kinetics in cultured cells. Nucleic Acids Res..

[66] Doucet AJ, Hulme AE, Sahinovic E, Kulpa DA, Moldovan JB, Kopera HC, Athanikar JN, Hasnaoui M, Bucheton A, Moran JV (2010). Characterization of LINE-1 ribonucleoprotein particles. PLoS Genet..

[67] Fusco D, Accornero N, Lavoie B, Shenoy SM, Blanchard JM, Singer RH, Bertrand E (2003). Single mRNA molecules demonstrate probabilistic movement in living mammalian cells. Curr. Biol..

[68] Bogerd HP, Wiegand HL, Hulme AE, Garcia-Perez JL, O'Shea KS, Moran JV, Cullen BR (2006). Cellular inhibitors of long interspersed element 1 and Alu retrotransposition. Proc. Natl Acad. Sci. USA.

[69] Zhou A, Hassel BA, Silverman RH (1993). Expression cloning of 2-5A-dependent RNAase: a uniquely regulated mediator of interferon action. Cell.

[70] Dong B, Niwa M, Walter P, Silverman RH (2001). Basis for regulated RNA cleavage by functional analysis of RNase L and Ire1p. RNA.

[71] Xiang Y, Wang Z, Murakami J, Plummer S, Klein EA, Carpten JD, Trent JM, Isaacs WB, Casey G, Silverman RH (2003). Effects of RNase L mutations associated with prostate cancer on apoptosis induced by 2′,5′-oligoadenylates. Cancer Res..

[72] Baumal R, Law J, Buick RN, Kahn H, Yeger H, Sheldon K, Colgan T, Marks A (1986). Monoclonal antibodies to an epithelial ovarian adenocarcinoma: distinctive reactivity with xenografts of the original tumor and a cultured cell line. Cancer Res..

[73] Wei W, Morrish TA, Alisch RS, Moran JV (2000). A transient assay reveals that cultured human cells can accommodate multiple LINE-1 retrotransposition events. Anal. Biochem..

[74] Clarke ML, Burton RL, Hill AN, Litorja M, Nahm MH, Hwang J (2010). Low-cost, high-throughput, automated counting of bacterial colonies. Cytometry A.

[75] Livak KJ, Schmittgen TD (2001). Analysis of relative gene expression data using real-time quantitative PCR and the 2(-Delta Delta C(T)) Method. Methods.

[76] Bertrand E, Chartrand P, Schaefer M, Shenoy SM, Singer RH, Long RM (1998). Localization of ASH1 mRNA particles in living yeast. Mol. Cell.

[77] Le Roy F, Bisbal C, Silhol M, Martinand C, Lebleu B, Salehzada T (2001). The 2-5A/RNase L/RNase L inhibitor (RLI) [correction of (RNI)] pathway regulates mitochondrial mRNAs stability in interferon alpha-treated H9 cells. J. Biol. Chem..

[78] Silverman RH, Cayley PJ, Knight M, Gilbert CS, Kerr IM (1982). Control of the ppp(a2′p)nA system in HeLa cells. Effects of interferon and virus infection. Eur. J. Biochem..

[79] Freeman JD, Goodchild NL, Mager DL (1994). A modified indicator gene for selection of retrotransposition events in mammalian cells. Biotechniques.

[80] Esnault C, Casella JF, Heidmann T (2002). A Tetrahymena thermophila ribozyme-based indicator gene to detect transposition of marked retroelements in mammalian cells. Nucleic Acids Res..

[81] Muckenfuss H, Hamdorf M, Held U, Perkovic M, Lower J, Cichutek K, Flory E, Schumann GG, Munk C (2006). APOBEC3 proteins inhibit human LINE-1 retrotransposition. J. Biol. Chem..

[82] Yoneyama M, Kikuchi M, Natsukawa T, Shinobu N, Imaizumi T, Miyagishi M, Taira K, Akira S, Fujita T (2004). The RNA helicase RIG-I has an essential function in double-stranded RNA-induced innate antiviral responses. Nat. Immunol..

[83] Schneider CA, Rasband WS, Eliceiri KW (2012). NIH Image to ImageJ: 25 years of image analysis. Nat. Methods.

[84] Morrison TB, Weis JJ, Wittwer CT (1998). Quantification of low-copy transcripts by continuous SYBR Green I monitoring during amplification. Biotechniques.

[85] Goodier JL, Zhang L, Vetter MR, Kazazian HH (2007). LINE-1 ORF1 protein localizes in stress granules with other RNA-binding proteins, including components of RNA interference RNA-induced silencing complex. Mol. Cell. Biol..

[86] Onomoto K, Jogi M, Yoo JS, Narita R, Morimoto S, Takemura A, Sambhara S, Kawaguchi A, Osari S, Nagata K (2012). Critical role of an antiviral stress granule containing RIG-I and PKR in viral detection and innate immunity. PLoS One.

[87] Ribet D, Harper F, Dupressoir A, Dewannieux M, Pierron G, Heidmann T (2008). An infectious progenitor for the murine IAP retrotransposon: emergence of an intracellular genetic parasite from an ancient retrovirus. Genome Res..

[88] Wreschner DH, McCauley JW, Skehel JJ, Kerr IM (1981). Interferon action–sequence specificity of the ppp(A2′p)nA-dependent ribonuclease. Nature.

[89] Floyd-Smith G, Slattery E, Lengyel P (1981). Interferon action: RNA cleavage pattern of a (2′-5′)oligoadenylate–dependent endonuclease. Science.

[90] Han JQ, Wroblewski G, Xu Z, Silverman RH, Barton DJ (2004). Sensitivity of hepatitis C virus RNA to the antiviral enzyme ribonuclease L is determined by a subset of efficient cleavage sites. J. Interferon Cytokine Res..

[91] Silverman RH, Skehel JJ, James TC, Wreschner DH, Kerr IM (1983). rRNA cleavage as an index of ppp(A2′p)nA activity in interferon-treated encephalomyocarditis virus-infected cells. J. Virol..

[92] Wreschner DH, James TC, Silverman RH, Kerr IM (1981). Ribosomal RNA cleavage, nuclease activation and 2-5A(ppp(A2′p)nA) in interferon-treated cells. Nucleic Acids Res..

[93] Matlik K, Redik K, Speek M (2006). L1 antisense promoter drives tissue-specific transcription of human genes. J. Biomed. Biotechnol..

[94] Nilsen TW, Baglioni C (1979). Mechanism for discrimination between viral and host mRNA in interferon-treated cells. Proc. Natl Acad. Sci. USA.

[95] Nilsen TW, Weissman SG, Baglioni C (1980). Role of 2′,5′-oligo(adenylic acid) polymerase in the degradation of ribonucleic acid linked to double-stranded ribonucleic acid by extracts of interferon-treated cells. Biochemistry.

[96] Zaccolo M, Pozzan T (2002). Discrete microdomains with high concentration of cAMP in stimulated rat neonatal cardiac myocytes. Science.

[97] Maitra RK, Li G, Xiao W, Dong B, Torrence PF, Silverman RH (1995). Catalytic cleavage of an RNA target by 2-5A antisense and RNase L. J. Biol. Chem..

[98] Carpten J, Nupponen N, Isaacs S, Sood R, Robbins C, Xu J, Faruque M, Moses T, Ewing C, Gillanders E (2002). Germline mutations in the ribonuclease L gene in families showing linkage with HPC1. Nat. Genet..

[99] Silverman RH (2003). Implications for RNase L in prostate cancer biology. Biochemistry.

[100] Madsen BE, Ramos EM, Boulard M, Duda K, Overgaard J, Nordsmark M, Wiuf C, Hansen LL (2008). Germline mutation in RNASEL predicts increased risk of head and neck, uterine cervix and breast cancer. PLoS One.

[101] Bartsch DK, Fendrich V, Slater EP, Sina-Frey M, Rieder H, Greenhalf W, Chaloupka B, Hahn SA, Neoptolemos JP, Kress R (2005). RNASEL germline variants are associated with pancreatic cancer. Int. J. Cancer.

[102] Kruger S, Silber AS, Engel C, Gorgens H, Mangold E, Pagenstecher C, Holinski-Feder E, von Knebel Doeberitz M, Moeslein G (2005). Arg462Gln sequence variation in the prostate-cancer-susceptibility gene RNASEL and age of onset of hereditary non-polyposis colorectal cancer: a case-control study. Lancet Oncol..

[103] Malathi K, Paranjape JM, Ganapathi R, Silverman RH (2004). HPC1/RNASEL mediates apoptosis of prostate cancer cells treated with 2′,5′-oligoadenylates, topoisomerase I inhibitors, and tumor necrosis factor-related apoptosis-inducing ligand. Cancer Res..

[104] Castelli JC, Hassel BA, Maran A, Paranjape J, Hewitt JA, Li XL, Hsu YT, Silverman RH, Youle RJ (1998). The role of 2′-5′ oligoadenylate-activated ribonuclease L in apoptosis. Cell Death Differ..

[105] Zhou A, Paranjape J, Brown TL, Nie H, Naik S, Dong B, Chang A, Trapp B, Fairchild R, Colmenares C (1997). Interferon action and apoptosis are defective in mice devoid of 2′,5′-oligoadenylate-dependent RNase L. EMBO J..

[106] Alvarez-Cubero MJ, Saiz M, Martinez-Gonzalez LJ, Alvarez JC, Lorente JA, Cozar JM (2012). Genetic analysis of the principal genes related to prostate cancer: a review. Urol. Oncol..

[107] Solyom S, Ewing AD, Rahrmann EP, Doucet T, Nelson HH, Burns MB, Harris RS, Sigmon DF, Casella A, Erlanger B (2012). Extensive somatic L1 retrotransposition in colorectal tumors. Genome Res..

[108] Lee E, Iskow R, Yang L, Gokcumen O, Haseley P, Luquette LJ, Lohr JG, Harris CC, Ding L, Wilson RK (2012). Landscape of somatic retrotransposition in human cancers. Science.

[109] Iskow RC, McCabe MT, Mills RE, Torene S, Pittard WS, Neuwald AF, Van Meir EG, Vertino PM, Devine SE (2010). Natural mutagenesis of human genomes by endogenous retrotransposons. Cell.

[110] Shukla R, Upton KR, Munoz-Lopez M, Gerhardt DJ, Fisher ME, Nguyen T, Brennan PM, Baillie JK, Collino A, Ghisletti S (2013). Endogenous retrotransposition activates oncogenic pathways in hepatocellular carcinoma. Cell.

