# Supplementary material for: RNase L restricts the mobility of engineered retrotransposons in cultured human cells
Source: Nucleic Acids Res. 2013 Dec 25;42(6):3803–20. doi: 10.1093/nar/gkt1308 (PMC3973342; doi:10.1093/nar/gkt1308)
Supplement: Supplementary Data [file supp_42_6_3803__index.html]

RNase L restricts the mobility of engineered retrotransposons in cultured human cells — RNase L restricts the mobility of engineered retrotransposons in cultured human cells — Supplementary Data 

# RNase L restricts the mobility of engineered retrotransposons in cultured human cells

## Supplementary Data

files

**Files in this Data Supplement:**

- Supplementary Data - pdf file
