# Supplementary material for: RNase L restricts the mobility of engineered retrotransposons in cultured human cells
Source: Nucleic Acids Res. 2013 Dec 25;42(6):3803–20. doi: 10.1093/nar/gkt1308 (PMC3973342; doi:10.1093/nar/gkt1308)
Supplement: Supplementary Data [file supp_gkt1308_nar-02586-h-2013-File011.pdf]

Supplementary Information

RNase L Restricts the Mobility of Engineered Retrotransposons in Cultured Human Cells

Ao Zhang[1,2], Beihua Dong[2], Aurélien J. Doucet[3], John B. Moldovan[4], John V. Moran[3,4,5,6], and Robert H. Silverman[1,2,*]

[1]Department of Molecular Medicine, Cleveland Clinic Lerner College of Medicine of Case Western Reserve University, and [2]Department of Cancer Biology, Lerner Research Institute, Cleveland Clinic, Cleveland, Ohio, 44195, USA; [3]Department of Human Genetics, [4]Cellular and Molecular Biology Program, [5]Department of Internal Medicine, and [6]Howard Hughes Medical Institute, University of Michigan Medical School, Ann Arbor, Michigan, 48109, USA

This file contains the following:

Supplementary Figure Legends.

Supplementary Figure 1. Representative flow cytometry plots of HeLa-M cells transfected with engineered human L1 constructs.

Supplementary Figure 2. Schematic of the siRNA-based L1 retrotransposition assay

Supplementary Figure 3. RNase L does not reduce HYG RNA expression from pIRESyg

Supplementary Figure 4. RNase L reduces L1 ORF1p expression from pDK500

Supplementary Figure 5. RNase L reduces L1 ORF2p expression from pAD500

Supplementary Figure Legends:

Supplementary Figure 1. *Representative flow cytometry plots of HeLa-M cells transfected with engineered human L1 constructs:* pLRE3-*mEGFPI* was co-transfected into cells with a pFLAG-CMV-2 empty vector, an amino-terminal FLAG-tagged RNase L expression construct (WT, R667A, or NΔ385), or an amino-terminal FLAG-tagged A3A (positive control) construct. For each sample, 20,000 cells were analyzed by FACS. The percentages of EGFP-positive cells are indicated on the plots. pJM111-LRE3-*mEGFPI* served as a negative control.

Supplementary Figure 2. *Schematic of the siRNA-based L1 retrotransposition assay:* Hey1b cells were transfected with a control siRNA pool or an siRNA pool that targets RNase L. The next day, the resultant cells were transfected with either pLRE3-*mEGFPI* or pJM111-LRE3-*mEGFPI*. After replating, one set of cells was used to monitor RNase L expression in western blot experiments. The other set of cells was subjected to puromycin selection for four days to enrich for cells containing the engineered L1 plasmids. The number of EGFP-positive cells was then determined by FACS.

Supplementary Figure 3. *RNase L does not reduce HYG RNA expression from pIREShyg:* HeLa-M cells were co-transfected with pIRESHyg and either a pFLAG-CMV-2 empty vector (vector control) or the indicated RNase L expression constructs (WT, R667A, or NΔ385). Levels of HYG RNA were determined 48 hours post-transfection using the Sybr Green method. The X-axis indicates the RNase L co-transfected samples. The Y-axis indicates the relative expression level of HYG RNA transcribed from pIRESHyg. The HYG RNA amounts were normalized with endogenous GAPDH levels. The primers used to detect HYG RNA were the same as in Figure 7 and described in Materials and Methods. The following primers were used to detect the endogenous GAPDH RNA: forward primer, 5'-GGAGCGAGATCCCTCCAAAAT-3'; reverse primer, 5'-GGCTGTTGTCATACTTCTCATGG-3'. Data are represented as the mean ± SD from three technical replicates of a single representative experiment. No statistically significant difference was found with one-way ANOVA and post-hoc tests. The experiment was conducted twice (separate biological replicates) with similar results.

Supplementary Figure 4. *RNase L reduces L1 ORF1p expression from pDK500:* (A) A schematic of the pDK500 construct is shown at the top of the Figure. The labels are the same as noted in Figure 1A. *(B) Results of Western blotting:* HeLa-M cells were co-transfected with pDK500 and either a pFLAG-CMV-2 empty vector or the indicated RNase L expression constructs (WT, R667A, or NΔ385). After four additional days of hygromycin selection, total cell lysates and RNP fractions were prepared for western blot analysis. L1 ORF1p was detected with an anti-T7 antibody. Endogenous ribosomal S6 protein served as a loading/transfer control. β-actin was used to distinguish the total cell lysate and RNP fractions. Shown are two different exposures of the ORF1p western blot. The experiments were repeated twice (biological replicates) with similar results. Depicted are the data from one experiment. Mock indicates untransfected cells.

Supplementary Figure 5. *RNase L reduces L1 ORF2p expression from pAD500: (A) Map of pAD500:* A schematic of the pAD500 construct is shown at the top of the Figure. The labels are the same as noted in Figure 1A. *(B) Results of Western blotting:* HeLa-M cells were co-transfected with pAD500 and either a pFLAG-CMV-2 empty vector or the indicated RNase L expression constructs (WT, R667A, or NΔ385). After four additional days of hygromycin selection, total cell lysates and RNP fractions were prepared for western blot analysis. L1 ORF2p was detected with an anti-TAP antibody. Endogenous ribosomal S6 protein served as a loading/transfer control. β-actin was used to distinguish the total cell lysate and RNP fractions. The experiments were repeated twice (biological replicates) with similar results. Depicted are the data from one experiment. Mock indicates untransfected cells.

Supplementary Figure 1.

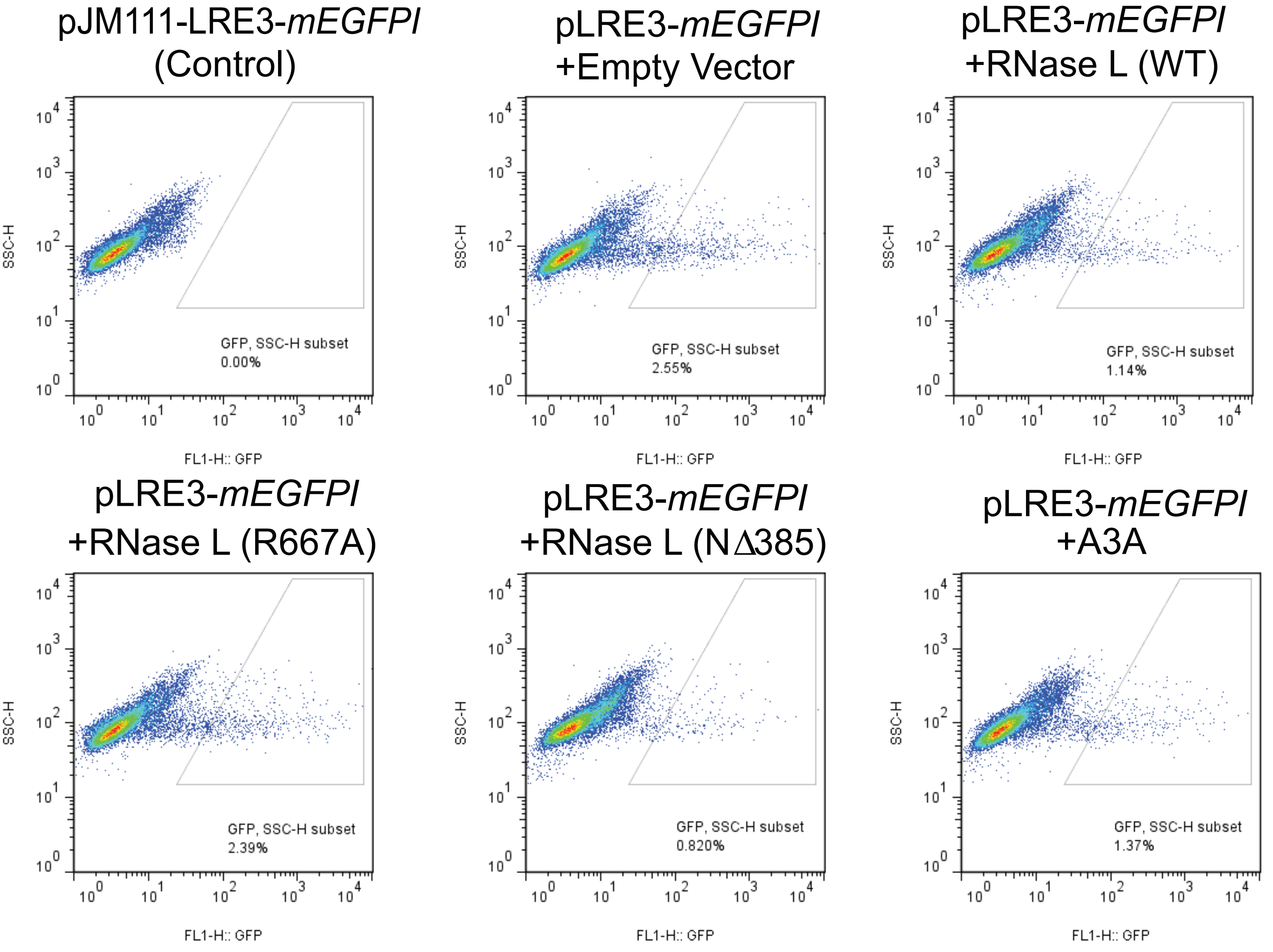

Supplementary Figure 2

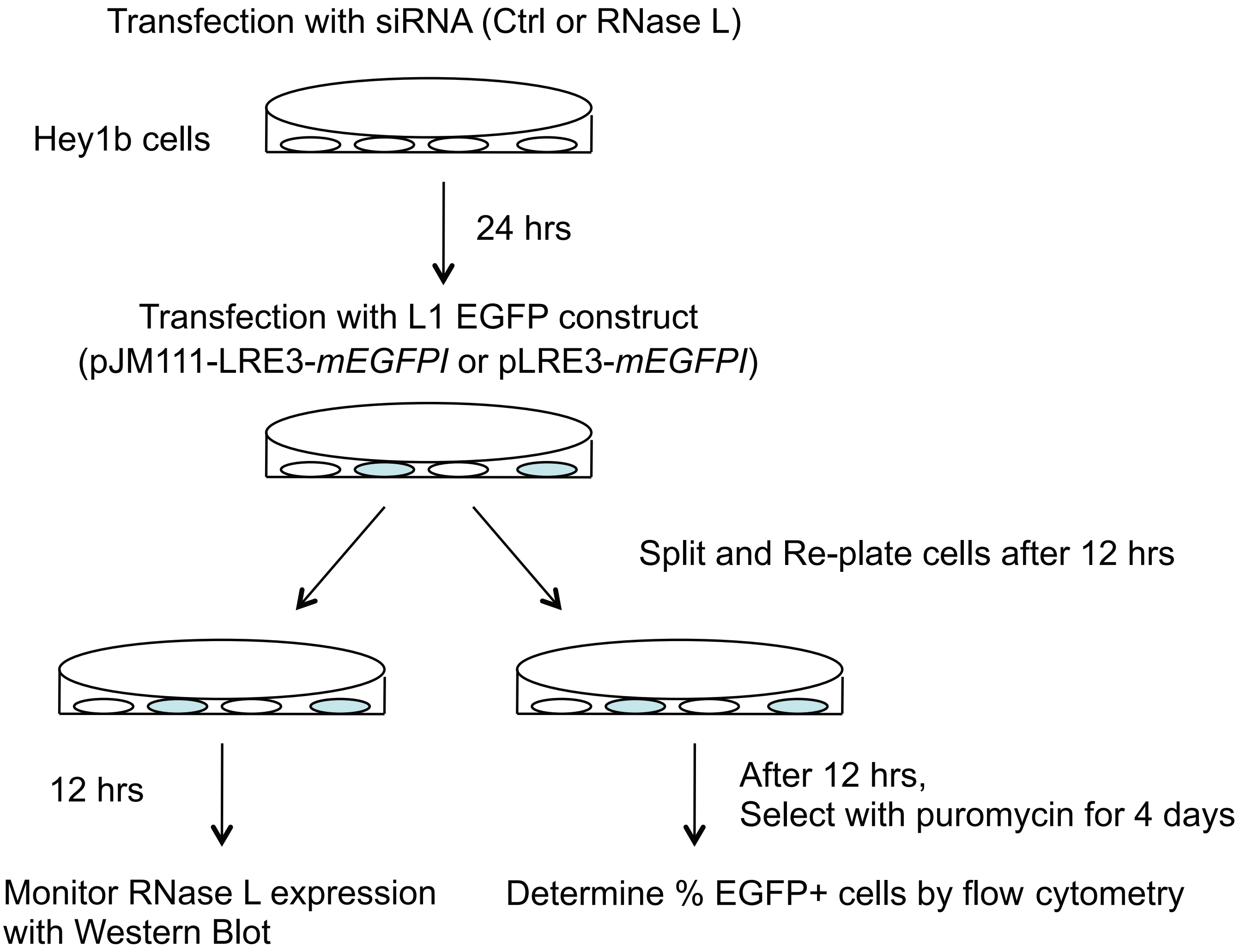

Supplementary Figure 3

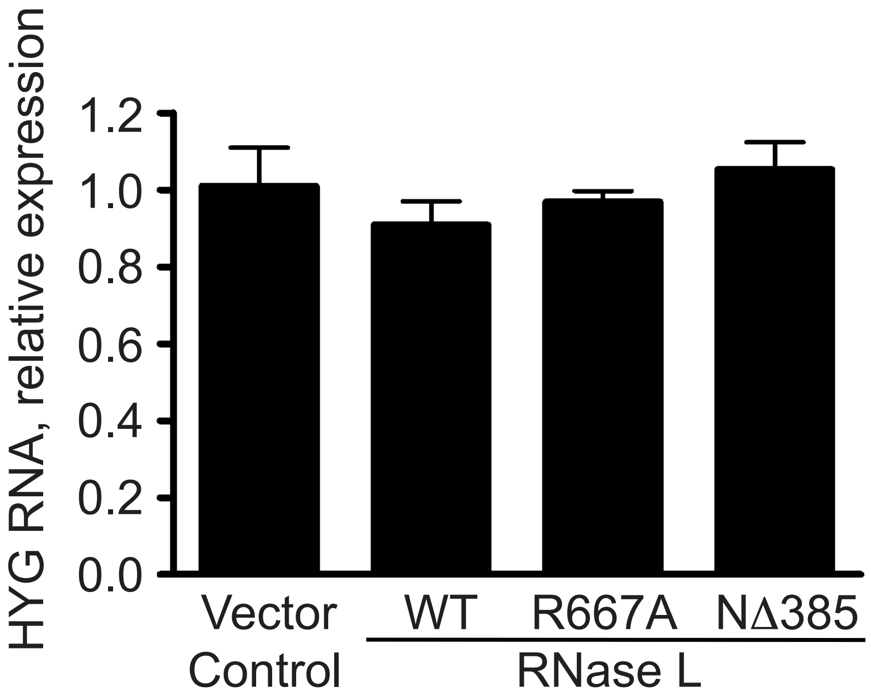

Supplementary Figure 4

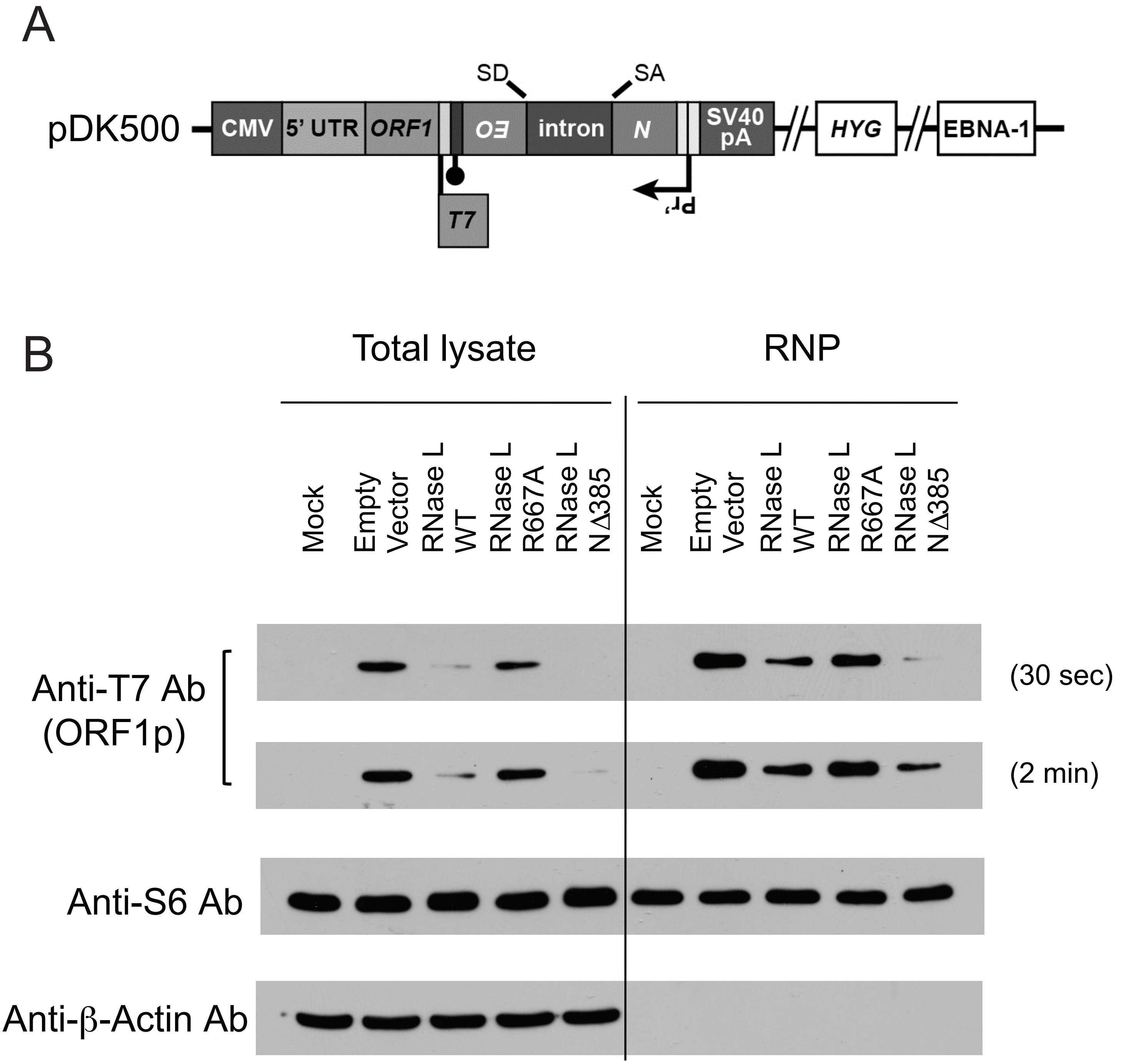

A

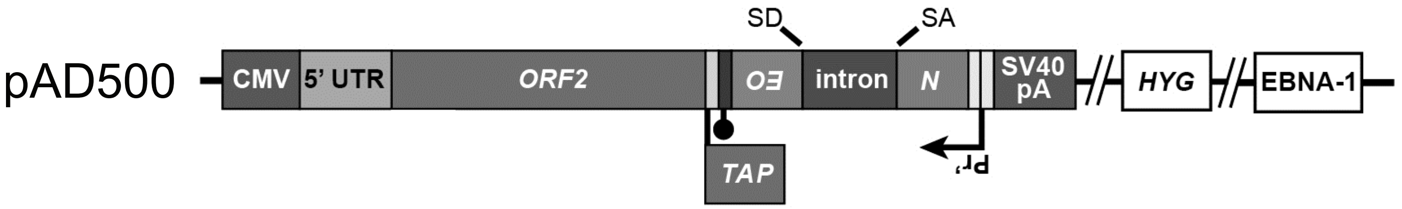
pAD500
CMV
5' UTR
ORF2
O3
intron
N
SV40 pA
HYG
EBNA-1
SD
SA
TAP
Pr

B

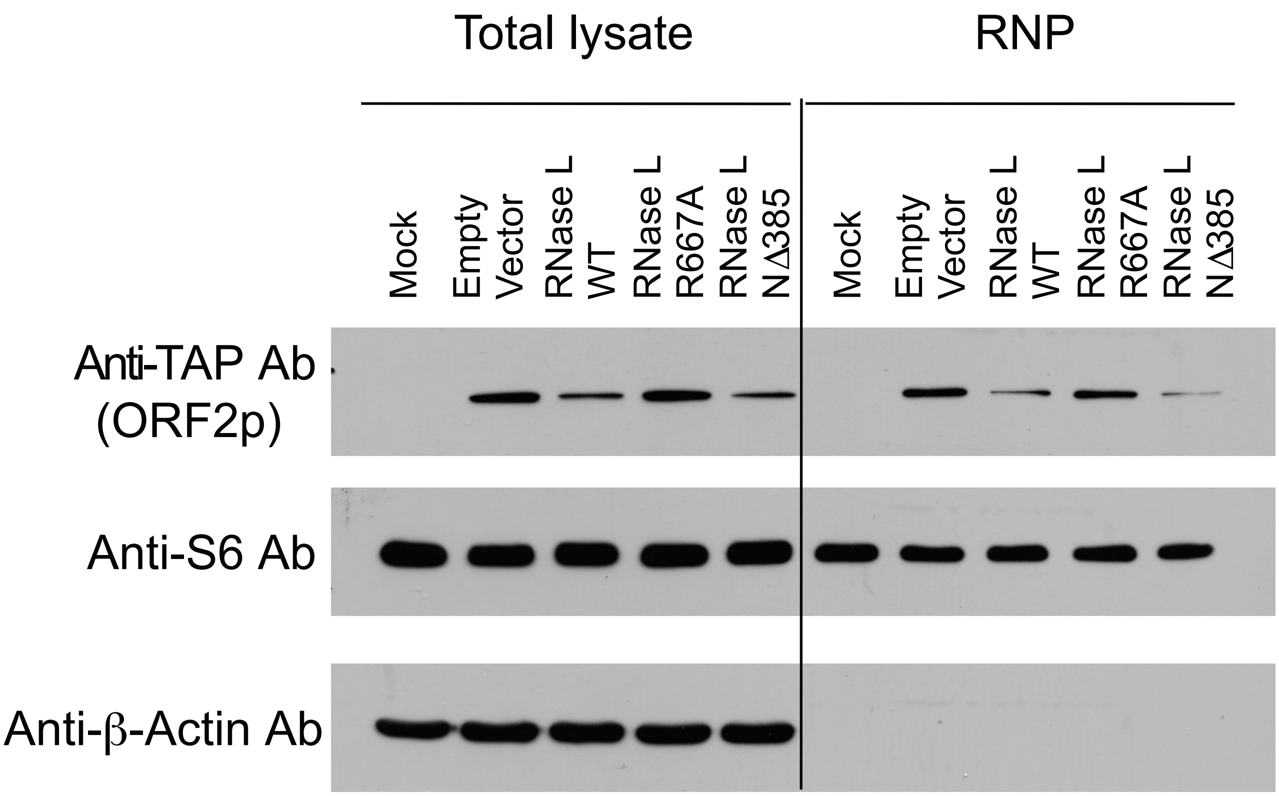
Total lysate
RNP
Mock
Empty Vector
RNase L WT
RNase L R667A
RNase L NΔ385
Mock
Empty Vector
RNase L WT
RNase L R667A
RNase L NΔ385
Anti-TAP Ab (ORF2p)
Anti-S6 Ab
Anti-β-Actin Ab
